# Cu-doped TiO_2_ nanoparticles improve local antitumor immune activation and optimize dendritic cell vaccine strategies

**DOI:** 10.1186/s12951-023-01844-z

**Published:** 2023-03-13

**Authors:** Evelien Hesemans, Neshat Saffarzadeh, Christy Maksoudian, Mukaddes Izci, Tianjiao Chu, Carla Rios Luci, Yuqing Wang, Hendrik Naatz, Sebastian Thieme, Cornelia Richter, Bella B. Manshian, Suman Pokhrel, Lutz Mädler, Stefaan J. Soenen

**Affiliations:** 1 NanoHealth and Optical Imaging Group, Department of Imaging and Pathology, KU Leuven, Leuven, Belgium; 2 Leibniz Institute for Materials Engineering IWT, Badgasteiner Straße 3, 28359 Bremen, Germany; 3 Faculty of Production Engineering, University of Bremen, Badgasteiner Straße 1, 28359 Bremen, Germany; 4 UniversitatsKlinikum, Dresden, Germany; 5 Translational Cell and Tissue Research Unit, Department of Imaging and Pathology, KU Leuven, Leuven, Belgium; 6 Leuven Cancer Institute, KU Leuven, Leuven, Belgium; 7 KU Leuven Institute of Physics-Based Modeling for In Silico Health, KU Leuven, Leuven, Belgium

**Keywords:** Nanomedicine, Dendritic cell vaccine, Tumor therapy, Metal (oxide) nanoparticles

## Abstract

Nanoparticle-mediated cancer immunotherapy holds great promise, but more efforts are needed to obtain nanoformulations that result in a full scale activation of innate and adaptive immune components that specifically target the tumors. We generated a series of copper-doped TiO_2_ nanoparticles in order to tune the kinetics and full extent of Cu^2+^ ion release from the remnant TiO_2_ nanocrystals. Fine-tuning nanoparticle properties resulted in a formulation of 33% Cu-doped TiO_2_ which enabled short-lived hyperactivation of dendritic cells and hereby promoted immunotherapy. The nanoparticles result in highly efficient activation of dendritic cells ex vivo, which upon transplantation in tumor bearing mice, exceeded the therapeutic outcomes obtained with classically stimulated dendritic cells. Efficacious but simple nanomaterials that can promote dendritic cancer cell vaccination strategies open up new avenues for improved immunotherapy and human health.

**Graphical Abstract:**

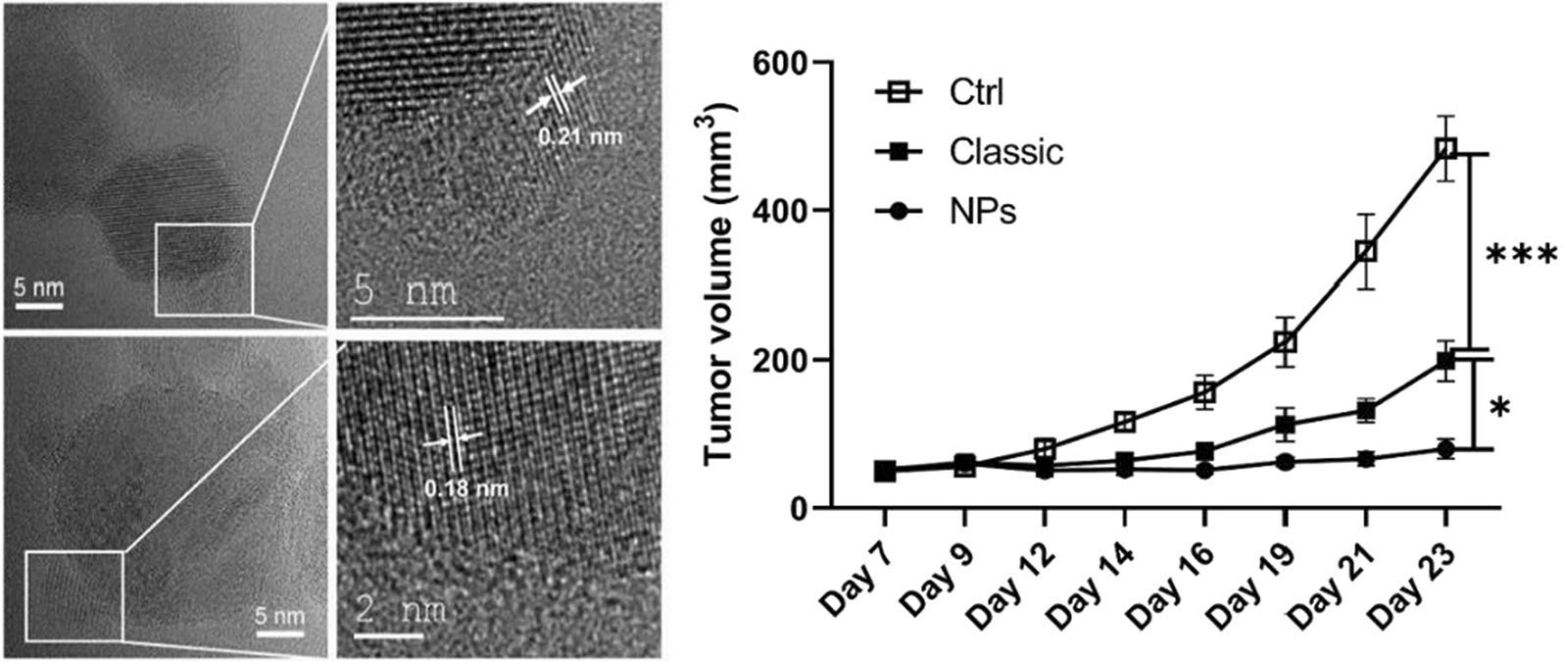

**Supplementary Information:**

The online version contains supplementary material available at 10.1186/s12951-023-01844-z.

## Introduction

The therapeutic use of engineered nanomaterials (NMs) has been a major focus of attention for nearly two decades [1]. In particular for organic NMs, such as lipid- or polymeric formulations that act as carriers for more common chemotherapeutics or genetic cargo (pDNA, mRNA, siRNA), (pre)clinical interest has been very high [2, 3]. The main advantages of organic NMs as carriers of common therapeutic agents lies in altering the bioavailability and pharmacokinetics of drugs that were already used in clinic [2]. The overall clinical translation has remained rather low, but the increase in other therapeutic research domains such as immunotherapy or small molecule discovery has opened the doors for more powerful applications of nanomedicines [1, 4]. Recent examples include of course the COVID-19 vaccines of Pfizer ^®^ and Moderna^®^, [5] which have demonstrated the potential of nanomedicines on a global scale to a broad audience.

Inorganic NMs have also been explored for biomedical use, where they are primarily employed as contrast agents for non-invasive imaging (*e.g*. iron oxide NMs as magnetic resonance imaging (MRI) probes) [6]. Their therapeutic use has remained more limited and is predominantly focused on their ability to act as mediators to convert external impulses (magnetic or optical) into heat and hereby serve as local heat sources for cancer hyperthermia [7, 8]. The unique physical and chemical characteristics of inorganic NMs can however bestow them with interesting therapeutic properties. Iron oxide NMs have, for example, been shown to result in macrophage polarization, where immunosuppressive (M2) tumor associated macrophages (TAMs) could be converted into inflammatory (M1) TAMs that had a direct effect on tumor growth [9]. These effects could be further exacerbated by combining iron oxide NMs with so-called immune checkpoint inhibitors that prevent tumor-mediated T cell suppression [10].

Other NMs that have obtained interesting preclinical results, are CuO-based NMs. Recent studies have indicated that pure CuO NMs significantly reduced tumor growth in pancreatic adenocarcinoma models and significantly increased the level of apoptosis in tumor-initiating cells [11]. As pure CuO tends to dissolve rather quickly and can result in imbalances in Cu^2+^ homeostasis in healthy organs, various methods have been looked into to try and affect the dissolution kinetics of the CuO NMs. One example lies in the inclusion of another metal as a dopant in the CuO NMs [12]. Upon using Zn-doped CuO NMs, significant therapeutic effects were observed in both glioblastoma as well as pancreatic tumor studies [13–15]. Interestingly, it was observed that the Zn-doped CuO NMs could resensitize glioblastoma cells to temozolomide, the most commonly used chemotherapeutic agent against glioblastoma [14]. In our own work, we have explored different levels of Fe as dopant in CuO NMs and observed significant cancer-selective toxicity in particular for 6% Fe-doped CuO NMs [12]. The selective toxicity was linked to the dissolution kinetics of the NMs, which decreased for higher levels of Fe-dopant. Interestingly, the use of 6% Fe-doped CuO NMs resulted in a pro-inflammatory effect that enhanced the effect of immunotherapeutic agents and even prevented relapse of the tumors in rechallenge experiments [12].

Copper-doped NMs have already shown great promise in their use as anticancer agents, and specifically as triggers for anti-cancer immune responses. In particular copper-cysteamine NMs have been studied in various tumor models and were found to induce potent antitumor immune responses via maturation of dendritic cells (DCs). This was followed by subsequent activation of T cells, which inhibited tumor growth by killing or suppressing tumor cells [16]. The copper-cysteamine NMs mainly act as stimulants that generate high levels of oxidative stress upon external stimulation by either light or X-rays [17, 18]. Here, we mainly were interested in looking at the specific contribution of Cu^2+^ ions, on immune cell activation and therefore doped the copper ions inside a stable TiO_2_ matrix, which is known not to dissolve in a physiological environment.

Much remains unknown about the specific therapeutic effects of CuO NMs, with regards to the specific contribution of metallic or ionic components. The use of dopants seems to play a major role in controlling the dissolution kinetics of the CuO NMs, and thus affects mainly the kinetics, but also in part the overall level of ionic Cu release. This in turn can trigger responses in a cellular environment, where high levels of Cu^2+^ ions activates heavy metal ATPases which reduce excess Cu^2+^ to Cu^+^. Toxicity can then occur in case the kinetics of Cu^2+^ release surpasses the ATPase reduction into Cu^+^, resulting in oxidative stress and possible cell death [19]. In the present work, we aimed to exploit Cu-mediated toxicity as a possible anticancer therapeutic by doping different levels of Cu^2+^ in a TiO_2_ matrix, which is less prone to dissolution. The efficacy of TiO_2_ NPs doped with 1, 5, 10, or 33% Cu for cancer therapy was investigated. We furthermore explored the different mechanisms by which any therapeutic effect elicited by the NMs could be maximized using appropriate combinatorial regimes.

## Results

### Nanoparticle synthesis and characterization

Flame spray pyrolysis was utilized for the production of pure TiO_2_, pure CuO and Cu-doped TiO_2_ nanoparticles, containing 1, 5, 10 or 33% Cu. Specific surface areas (SSA) obtained from BET measurements showed relatively high SSA (larger than 100 m^2^/g) for pure TiO_2_ and Cu doped TiO_2_ compared to pure CuO (67.294 m^2^/g). At lower Cu dopings (1%, 5% and 10%), the SSA of pure TiO_2_ increased by about 10%. It is known that the small particles deposited/doped on the large support increases the SSA of the overall material and inhibits the growth [20, 21]. Thus, the primary particle size decreased due to doping of Cu in TiO_2_. For high copper concentration, due to the formation of CuO particles which were similar in size than TiO_2_ particles, the specific surface area was slightly lower compared with lower copper dopings, but higher than pure CuO resulting from the functionalization of small particles. The primary particle sizes of all materials under investigation were in the range of 12–14 nm.

**Fig. 1:**
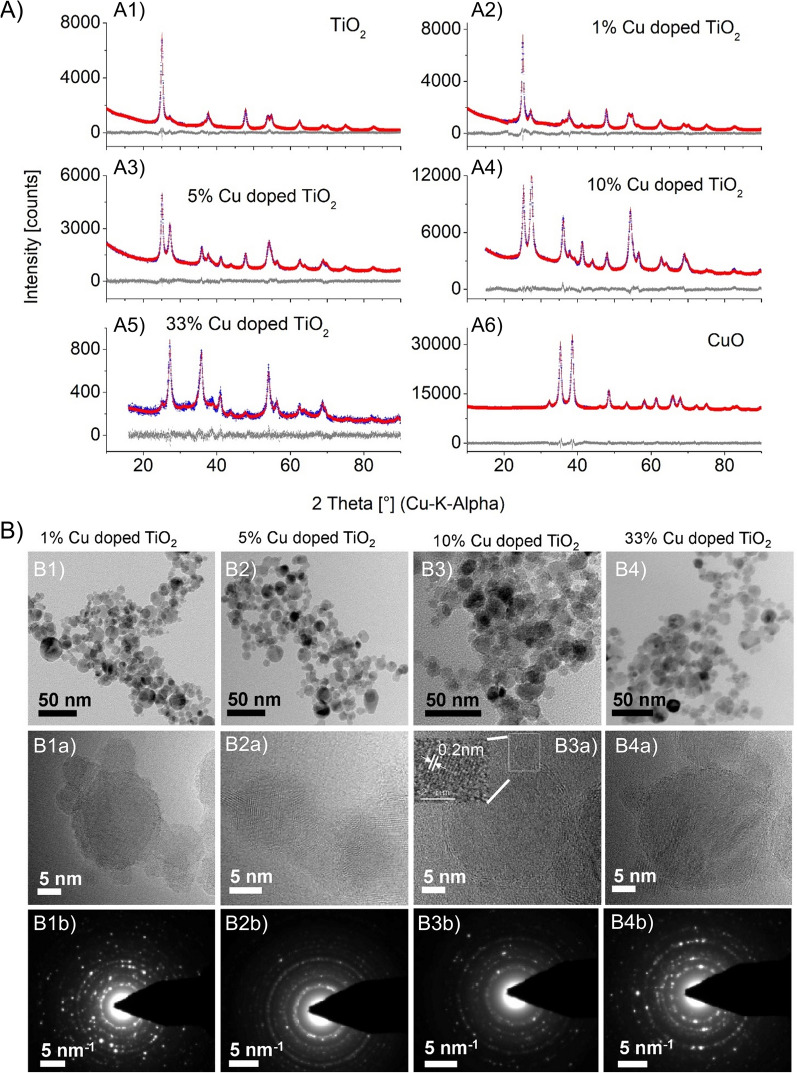
**A** XRD patterns of pure A1) TiO_2_, A2) 1%, A3) 5%, A4) 10%, A5) 33% Cu doped TiO_2_ and A6) pure CuO. **B** Low resolution TEM images (B1-B4), which indicate spherical morphology of as prepared samples of B1) 1%, B2) 5%, B3) 10% and B4) 33% Cu-doped TiO_2_ NPs with primary particle sizes in the range of 10–20 nm. Images B1a-B4a (middle row) display HR-TEM images of 1%, 5%, 10%, 33% Cu doped TiO_2_ and images B1b-B4b display SAED patterns (third row). Data shows particles are highly crystalline and ultrafine

The X-ray diffraction (XRD) patterns of the as prepared samples are shown in Fig. 1A, while the corresponding compositions and crystallite sizes are listed in Table 1.

**Table 1:** Specific surface area, primary particle, density, crystallite size and phase composition of pure TiO_2_, pure CuO and Cu-doped TiO_2_ nanoparticles

Sample	Density (Rietveld analysis), (g/cm^3^)	Specific Surface Area (SSA), (m^2^/g)	Phase composition			*d*_BET,_ anatase (nm)	*d*_XRD,_ anatase (nm)	*d*_XRD,_ rutile (nm)	*d*_XRD,_ tenorite (nm)
			Anatase (%)	Rutile (%)	Tenorite (%)				
TiO_2_	3.98	107.4 ± 3.1	77.3	22.7	0	13.6	24.8	*	-
1% Cu/TiO_2_	3.99	120.7 ± 0.9	76.5	23.5	0	12.4	28.4	11.1	-
5%Cu/TiO_2_	4.10	113.3 ± 8.0	45.7	54.3	0	12.1	20.9	11.2	-
10%Cu/TiO_2_	4.23	115.4 ± 2.8	21.7	78.3	0	12.0	8.5	13.1	-
33%Cu/TiO_2_	4.07	97.0 ± 9.0	5.4	81.8	12.8	13.9	12.9	12.5	14.2
CuO	6.52	65.2 ± 2.1	0	0	100	13.7	–	–	15.6

^*^represents crystallite size of rutile in pure TiO_2_ which was not refined due to low rutile mass

Pure TiO_2_ contained 77.28% anatase and 22.72% rutile, typical for flame-made TiO_2_. The increasing intensity of the reflections at 27.32°, 36.09°, 41.22°, 54.28° indicated that addition of copper increased rutile molar percentage. During the gas phase doping, Cu replaced Ti in the TiO_2_ lattice leading to higher number of defects and/or oxygen vacancies within the anatase phase resulting in faster formation and growth of rutile [22, 23]. The CuO and/or Cu_2_O phase were not detected in Cu-doped TiO_2_ at Cu-doping of 1%, 5% and 10%. A similar observation was reported by Teleki et al*.* where the copper phase in FSP- prepared Cu doped TiO_2_ nanoparticles were absent [21].

Low-resolution transmission electron microscopy (Fig. 1B, first row), revealed that FSP-prepared 1%, 5%, 10% and 33% Cu-doped TiO_2_ were spherical nanoparticles with primary particle sizes in the range of 10–20 nm and exhibited indistinguishable crystalline morphology. The highly crystalline nature of these particles was evidenced based on the distinct SAED crystallographic rings (Fig. 1B, third row). In the high-resolution TEM images (Fig. 1B, middle row), small particles (particle sizes less than 5 nm) decorated on large particles were observed, a common phenomenon for noble metal doped nanoparticles. Contrary to the observation, transition metals such as Cu are expected to substitute Ti and dope the parent matrix instead of nucleating as a metal on the surface.

### Analysis of Cu oxidation states

**Fig. 2:**
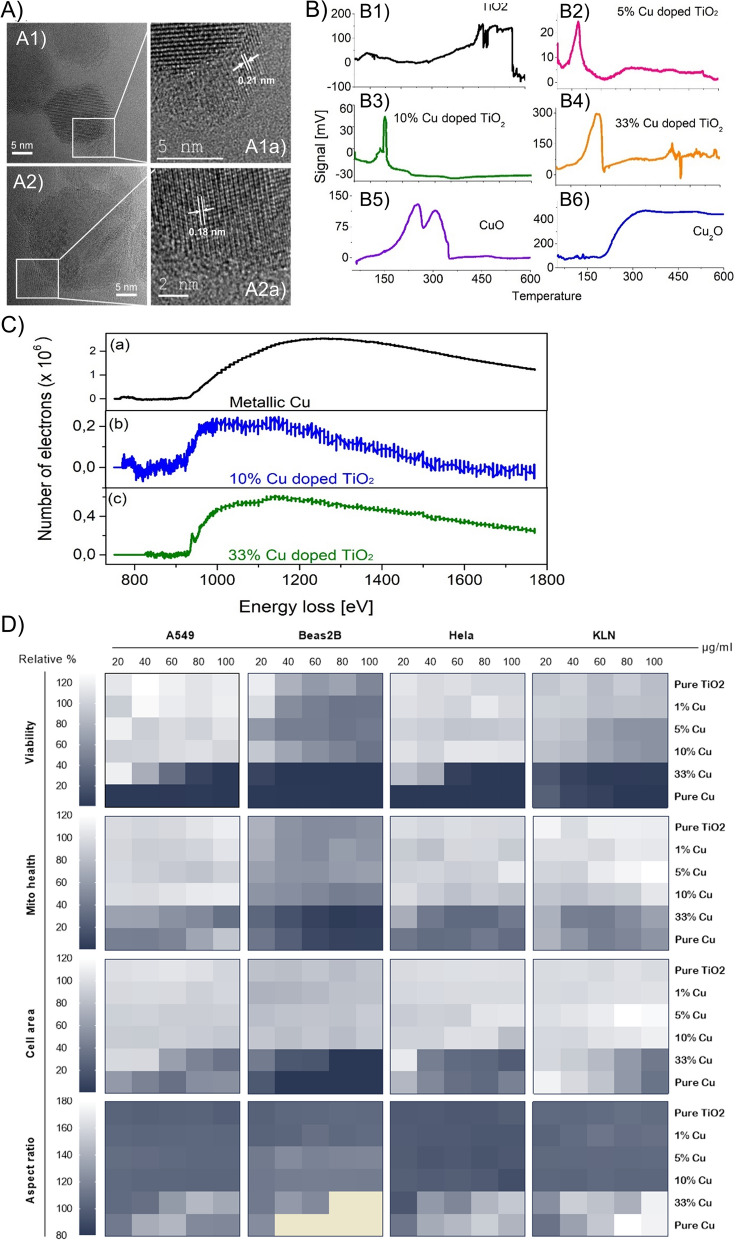
**A** HR-TEM images of Cu-doped TiO_2_ nanoparticles with lattice distance in the range of 0.18 to 0.21 nm. A1 and A2 show the HR-TEM and the area indicated with a white rectangle is shown as a zoomed in image in A1a and A1b, respectively. **B** TPR diagrams of pure B1) TiO_2_, B2) 5%, B3) 10%, B4) 33% Cu doped TiO_2_, B5) CuO and B6) Cu_2_O Pure CuO shows two reduction signals around 240 °C and 310 °C which were attributed to reduction of Cu^2+^/Cu^+^ and Cu.^+^/Cu0, respectively. Each Cu doped TiO_2_ display one peak indicating the presence of Cu_2_O on the surface. **C** electron energy loss spectrocopy (EELS spectra) of (a) pure Cu measured using copper metal as an instrument standard (b) 10% Cu doped TiO_2_ and (c) 33% Cu doped TiO_2_. In all the three samples, the signals occur very similarly as metallic Cu. Hence, the small particles homogenously distributed on the TiO_2_ matrix is concluded as metallic copper. **D** Representative heat map displaying the effect of the different NPs on the viability, cell area, cell aspect ratio and mitochondrial health of human lung adenocarcinoma (A549), human bronchoepithelial cells (Beas2B), human cervical tumor cells (HeLa) and murine lung adenocarcinoma (KLN 205)

The CuO nanoparticles obtained by FSP are usually around 12-14 nm in size (Table 1) but the small particles here are less than 5 nm. To find out the oxidation states of the Cu in those NPs, lattice distances were measured by probing these particles in the HRTEM imaging. Considering 10% Cu doped TiO_2_, the 0.2 nm lattice distances of the small particles reasonably agree with metallic copper (Fig. 1B3a, inset of the High resolution image of 10% Cu doped TiO_2_). Such metallic copper was also observed in 5%, 33% Cu doped TiO_2_ where the lattice distances were in the range of 0.18 to 0.21 nm (see Fig. 2A). Apart from the metallic copper, the lattice distances matching CuO and Cu_2_O were also observed in the doped particles. To conclusively determine the Cu oxidation state in the doped particles, EELS analysis showed the formation of L_2,3_ edge due to the excitation of 2p electrons to the vacant 3d states localized at Cu particles. The L_3_ edge resulted from the transition from the 2p_3/2_ level while the L_2_ edge is from the 2p_1/2_ level by the spin–orbit splitting. Pure Cu was measured as reference and smooth flat broad L_2,3_ edge was obtained without sharp peaks due to full occupancy of its 3d states in the range of 900 to 1000 eV. A sharp L_2,3_ edge was absent in the EELS diagram of 10% Cu doped TiO_2_. Hence, the results indicate the Cu was in the form of metallic copper (Fig. 2C)).

An EELS signal at 935 eV for 33% Cu doped TiO_2_ indicated the presence of copper oxide as reported in the literature [24]. In the EELS spectra of the Cu^+^ case, apart from the L_2,3_ edge, an extra signal at 947.5 eV was observed. This extra signal is attributed to the transitions into the Cu 4 s states above the Fermi level. However, it is still unclear whether the Cu in the 33% Cu doped TiO_2_ is CuO or Cu_2_O. To confirm the redox state of the copper in the doped particles, temperature programmed reduction of pure CuO was performed. The data showed two signals at 240 °C and 310 °C attributed to the reduction of Cu^2+^/Cu^+^ and Cu^+^/Cu^0^ couples, respectively. Such reduction signals were absent prior to 400 °C for pure TiO_2_. Hence, in all the doped samples, the signals represents basically metallic Cu and reduced Cu_2_O (Fig. 2B, C).

### NP cytotoxicity and potential cancer-cell selective toxicity

Next, we aimed to study the efficacy of the different Cu-doped TiO_2_ NPs at killing cancer cells and investigate whether there is a difference in sensitivity between cancerous and non-cancerous cell types. As a representative panel human lung adenocarcinoma cells (A549), murine lung squamous carcinoma cells (KLN 205), and human bronchial epithelial cells (Beas-2B) were used and exposed to the different NPs at concentrations ranging from 20 to 100 µg/ml. Figure 2D, Additional file 1: Figure S1 reveals clear differences between NP formulations, concentrations and cell types. For all the cell types, low levels of Cu^2+^-ions in the TiO_2_ NPs does not seem to have any effect on NP cytotoxicity, but 33% Cu-doped TiO_2_ NPs resulted in clear concentration-dependent toxicity for all cell types. Pure CuO NPs resulted in high cytotoxicity, resulting in nearly complete cell death for Beas-2B and A549 cells at 20 µg/ml. While KLN-205 cells on the other hand, were less affected to CuO NPs than Beas-2B and A549 cells, 33% Cu-doped TiO_2_ NPs still resulted in significant loss of viability. A549 and Beas-2B cells displayed more resistance against 33% Cu-doped TiO_2_ NPs than to pure CuO NPs up to 40 µg/ml and showed significant cytotoxicity shortly after that. Nanoparticle-induced sensitivity to the different NPs was therefore clearly dependent on the cell type itself. This is in line with other reports [12, 25], where variations in cellular responses to NP-induced toxicity is influenced by a variety of intrinsic factors. These include, among others: the cellular defense mechanisms against oxidative stress, which may makes cells more or less responsive against NP-elicited reactive oxygen species (ROS) [26, 27]. Another factor constitutes the size of the cells themselves, where bigger cells would be more prone to interact with higher levels of the NMs as these tend to be colloidally less stable and would sediment on top of the cell surface during the incubation period [28].

Apart from the cell-dependent differences in sensitivity to the NPs, the intrinsic variations in chemical composition of the NPs also plays a major role. Pure CuO NPs are known to release Cu^2+^ ions which by activation of heavy metal ATPase can result in ROS and conversion into Cu^+^. While the toxicity of Cu is related to its redox species, the differential toxicity of Cu^2+^ and Cu^+^ remains poorly understood. A recent environmental study on the effects of Cu^2+^ and Cu^+^ on phytoplankton demonstrated that Cu^+^ played the major role in toxicity following its biotransformation from Cu^2+^ into Cu^+^.[29] In mammalian systems, Cu^+^ cannot bind proteins and enzymes, but it can also lead to ROS by generation of potent hydroxyl radicals [30]. The EELS and temperature-driven reduction experiments revealed that 33% Cu-doped TiO_2_ NPs contain both metallic Cu as well as Cu_2_O, the reduced form of CuO, which will result in Cu^+^ release. Therefore, for 33% Cu-doped TiO_2_ NPs, the contribution of Cu^2+^ to the observed toxicity will be negligible, suggesting that metallic Cu or Cu^+^ in itself is highly toxic. Taken into account that the NPs were given at the same weight, the main component (67%) of 33% Cu-doped NPs consisted out of the TiO_2_ matrix. Therefore, the level of copper ions at the same mass for 33% Cu-doped TiO_2_ NPs will be lower than those for pure CuO. Based on the EELS and temperature-driven reduction experiments, we therefore hypothesize that the level of Cu^+^ released by 33% Cu-doped TiO_2_ NPs is lower than the Cu^2+^ levels that are obtained for pure CuO NPs. These data suggest that Cu^+^ may therefore also be highly toxic for mammalian systems, similarly as observed for the phytoplankton.

When looking into the specific mechanisms underlying NP toxicity, in particular for the pure CuO NPs and 33% Cu-doped TiO_2_ NPs, cell viability is significantly reduced at relatively low NP concentrations. The loss of cell viability was furthermore linked to a loss in mitochondrial health, a reduction in cell spreading and an increase in cellular rounding (an increased aspect ratio). Given that the loss of viability occurred at lower concentrations than any of these secondary parameters, as well as the variability typically observed between different cell types, it remains unclear whether these were secondary effects linked to cell death itself, or whether these were the primary causes instigating cell death. Surprisingly, ROS generation was found to be relatively low, as shown for both A549 and KLN cells (Additional file 1: Figure S2). While a concentration-dependent increase in ROS was observed, this did not seem sufficiently strong to result in loss of cell viability. This was further evaluated by exposing the cells to the NPs in the presence of N-acetyl cysteine (NAC), a ROS scavenger, which had no effect on cell viability (Additional file 1: Figure S2), while reducing the observed ROS increase to near control levels (Additional file 1: Figure S2).

### Anti-tumoral effects of Cu-doped TiO_2_ NPs

**Fig. 3:**
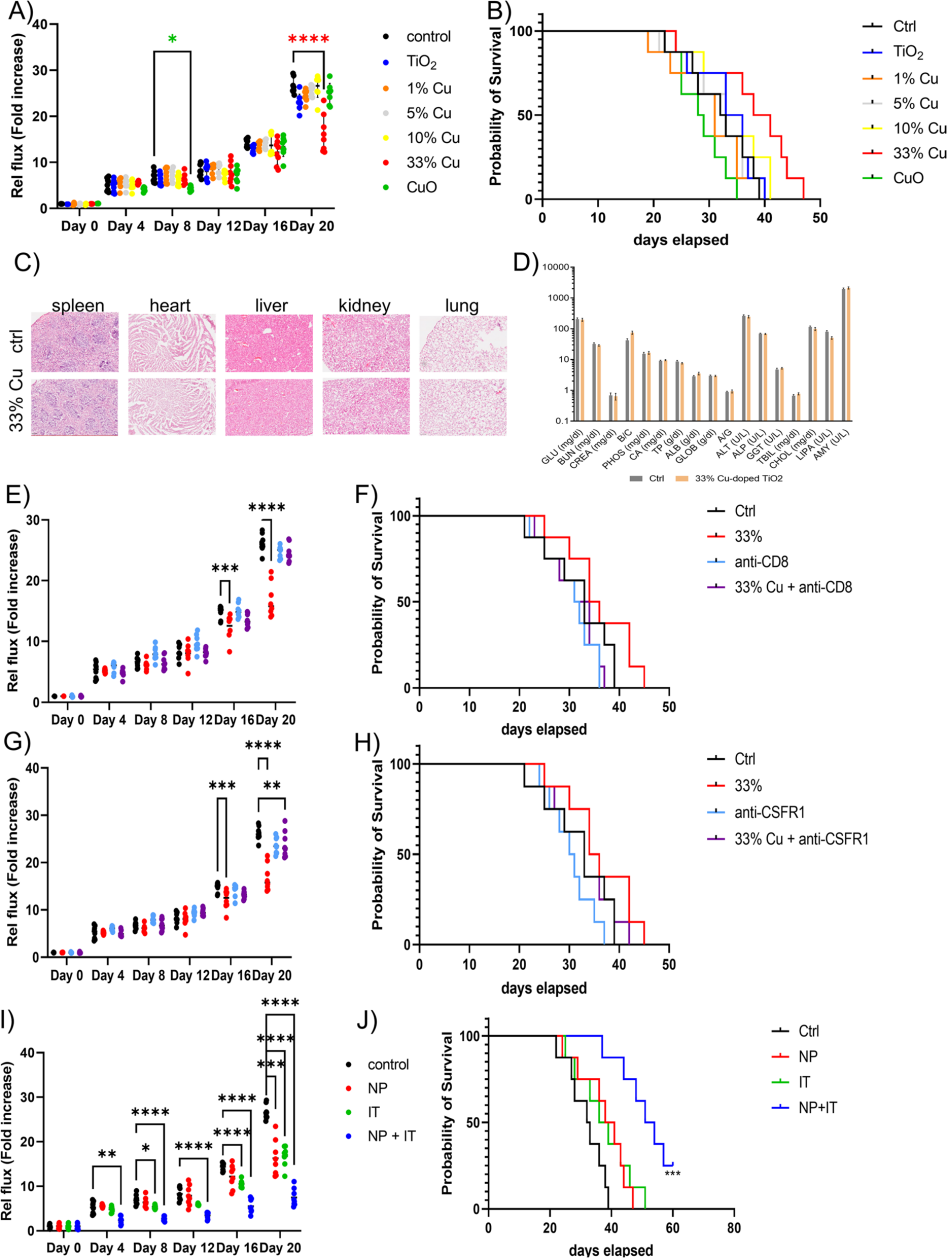
**A** Relative photon flux of luminescent KLN 205 tumors grafted subcutaneously treated with the respective NPs (100 µg/mouse, single bolus administered peritumorally) or vehicle control expressed as fold-difference compared to the original time point (day 0). **B** Kaplan-Meyer survival curves of the animals bearing subcutaneous KLN 205 tumors and treated with the respective NPs or vehicle control. **C** Representative micrographs of hematoxylin and eosin stained tissue sections obtained from different organs of control animals or animals given 33% Cu-doped TiO_2_ NPs. **D** Blood biochemistry analysis of blood samples obtained from tumor-bearing DBA2 mice having received saline (control) or 33% Cu-doped TiO_2_ NPs. **E** Relative photon flux expressed as fold difference and **F** Kaplan-Meyer survival curves of DBA2 mice bearing KLN 205 tumors treated with 33% Cu-doped TiO_2_ NPs, anti-CD8 antibody, combination of both or vehicle control (saline). The anti-CD8 antibody was administered intravenously in 3 different boluses each at 200 µg/mouse at 5, 3 and 1 day prior to NP or PBS administration. **G** Relative photon flux expressed as fold difference and **H** Kaplan-Meyer survival curves of DBA2 mice bearing KLN 205 tumors treated with 33% Cu-doped TiO_2_ NPs, anti-CSF1R antibody, combination of both or vehicle control (saline). The anti-CSFR1 antibody was administered intravenously in 3 different boluses each at 200 µg/mouse at 5, 3 and 1 day prior to NP or PBS administration. **I** Relative photon flux expressed as fold difference and **J** Kaplan-Meyer survival curves of DBA2 mice bearing KLN 205 tumors treated with 33% Cu-doped TiO_2_ NPs, anti-PD1 antibody as immunotherapy, combination of both or vehicle control (saline). The anti-PD1 antibody was administered intravenously in 3 different boluses each at 200 µg/mouse at 2 days prior to, together with or 2 days following NP or PBS administration. Significant differences between a treated group and untreated controls at the same time point are indicated where relevant (p < 0.05: *, p < 0.01: **; p < 0.001: ***; p < 0.0001: ****) based on ANOVA testing using GraphPad Prism 9 (*n* = 8)

As the in vitro data did not reveal a distinct cancer cell-specific cytotoxic effect, a relatively low dose of the different NMs was initially tested in KLN-205 tumor bearing DBA2 mice, being 100 µg/mouse. In line with the in vitro data, only minor insignificant effects were observed for pure TiO_2_ NPs, or for 1, 5 or 10% Cu-doped TiO_2_ NPs (Fig. 3A). There were significant reductions in tumor growth observed for both 33% Cu-doped TiO_2_ NPs as well as for pure CuO. However, for pure CuO NPs, these effects were only transient and resulted in negligible overall enhanced therapeutic effect at later time points. Furthermore, CuO NPs resulted in insignificant increases on animal survival. For 33% Cu-doped TiO_2_ NMs, the effects were only observed at a later stage, where the tumors appeared to be growing slower than for any other condition, but this only became significant after 16 days. Overall, the 33% Cu-doped TiO_2_ NPs had a minor improvement on animal survival (Fig. 3B). Microscopic analysis of hematoxylin and eosin-stained tissue sections of the major organs, did not reveal an differences between 33% Cu-doped TiO_2_-treated animals and control animals (Fig. 3C). Blood biochemistry analysis however revealed a transient but insignificant increase in some liver-related parameters for 33% Cu-doped TiO_2_, while this was more pronounced for pure CuO NPs (Fig. 3D). The influence of doping and the amount of Cu^2+^ released therefore play an important role in the safety and toxicity of the NMs, which is in line with previous studies, where pure ZnO and CuO NPs were found to be far more toxic than their doped counterparts, which often prevented their full use without undesired side-effects [12, 25, 31].

As the 33% Cu-doped TiO_2_ NPs resulted in a transient reduction in tumor growth without any observed off-target toxicity, this formulation was further studied in more detail as a potential therapeutic agent. In a first trial, the 33% Cu-doped TiO_2_ NPs were again given to DBA2 mice bearing a syngeneic KLN205 tumor. In this experiment, the NPs were provided either on their own, or in combination with anti-CD8 antibody or anti-CSFR antibody and compared to the effect these antibodies have on tumor growth as monotherapies. In both cases, the use of the antibodies resulted in a near complete inhibition of 33% Cu-doped TiO_2_-mediated therapeutic effect (Fig. 3E–H). The data reveal the importance of both the adaptive (CD8^+^ T cells) and innate (CSFR1^+^ macrophages) immune system on the therapeutic effect of the NMs.

To further confirm the contribution of the immune system on potentiating the therapeutic effect of 33% Cu-doped TiO_2_ NPs, the NPs were also tested together with anti-PD1 antibody, a clinically used immune checkpoint inhibitor. Interestingly, while the use of immune checkpoint inhibition on its own had only minimal effects, together with the 33% Cu-doped TiO_2_ NPs this resulted in a significant and prolonged reduction of tumor growth (Fig. 3I) and improved survival of tumor-bearing animals (Fig. 3J).

### Immunomodulatory effects of Cu-doped TiO_2_ NPs

**Fig. 4:**
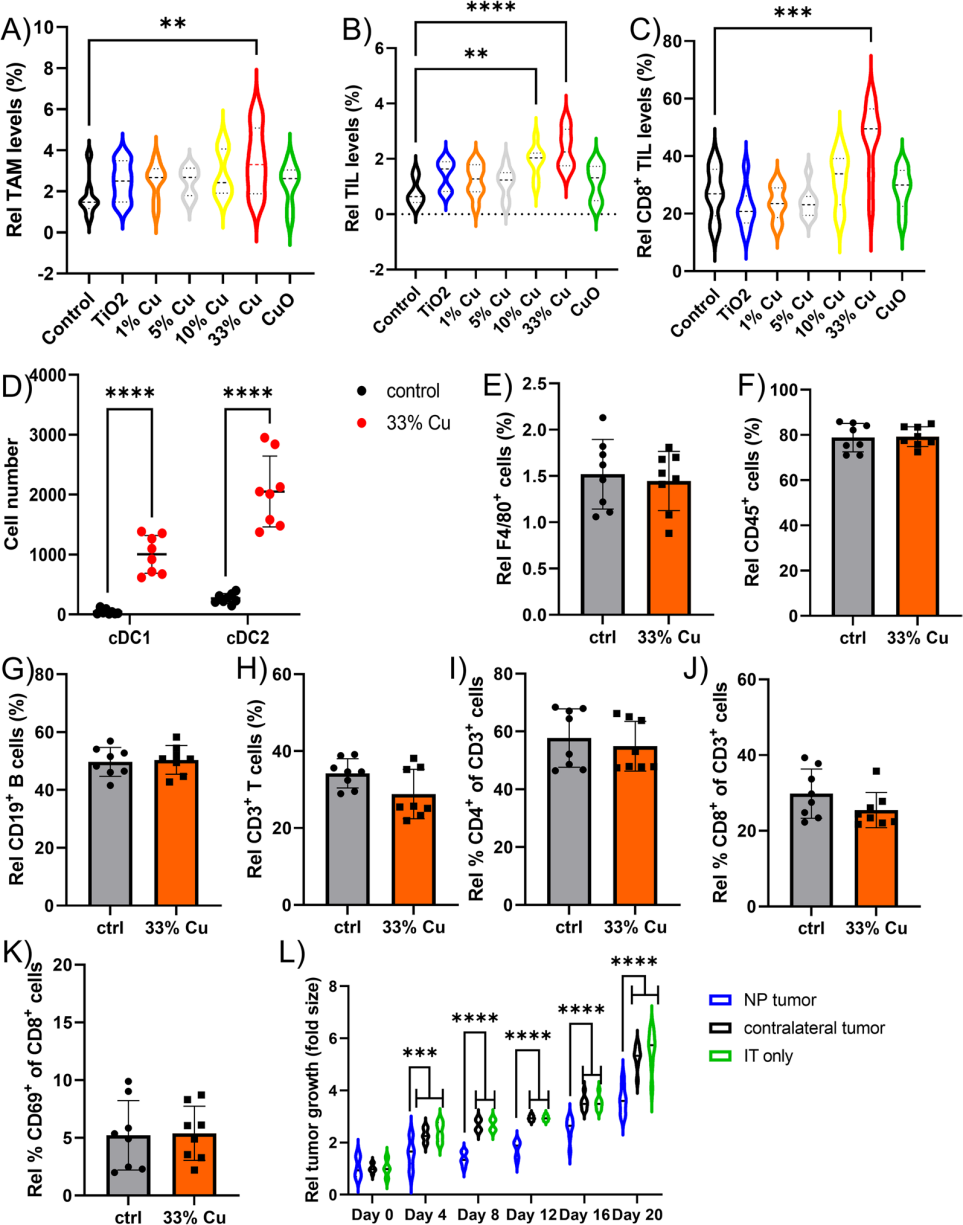
**A**–**C** Violin plots indicating the level of A) TAMs B) TILs, and C) CD8^+^ TILs expressed relative to the total number of A,B) all cells or C) CD3^+^ TILs as determined by ImageStreamX Mark II analysis (*n* = 8). **D** The number of conventional DCs (XCR1^+^ cDC1 or CD172α^+^ cDC2) observed in the tumor draining lymph node of KLN 205 tumors treated with vehicle (saline control) or 33% Cu-doped TiO_2_ NPs. **E–K** Histograms representing the relative percentage of E) F4/80^+^ macrophages, F) CD45^+^ lymphocytes, G) CD19^+^ B cells, H) CD3^+^ T cells, I) CD4^+^ T cells, J) CD8^+^ T cells, K) CD69^+^ T cells determined by ImageStreamX Mark II analysis of isolated spleens and expressed relative to E–H) total splenocytes, I,J) total CD3^+^ T cells, K) total CD8^+^ CD3^+^ T cells. **L)** Violin plots indicating the fold difference in tumor volumes as determined by caliper measurements in animals bearing 2 subcutaneous tumors on contralateral sides. The animals either systemically received anti-PD1 only (IT group, 200 µg/mouse administered 2 days before, together with or 2 days following saline administration), or received anti-PD1 systemically (200 µg/mouse administered 2 days before, together with or 2 days following NP administration) and the tumor on the right received a single bolus of 33% Cu-doped TiO_2_ NPs (NP group) versus vehicle control (saline) in the left tumor (contralateral group). Significant differences between a treated group and untreated controls at the same time point are indicated where relevant (p < 0.01: **; p < 0.001: ***; p < 0.0001: ****) based on ANOVA testing using GraphPad Prism 9 (*n* = 8)

The immunological contribution to the therapeutic efficacy of the Cu-doped TiO_2_ NPs was then studied by analyzing the immunological composition of the tumor microenvironment (TME) for all animals treated with the various NPs at 14 days post NP administration. Image-based flow cytometry data revealed low levels of tumor infiltrating lymphocytes (TILs) and tumor-associated macrophages (TAMs) under normal conditions, and these were minimally affected by most formulations (Fig. 4A, B). However, 10% Cu-doped TiO_2_ did increase TIL levels, while 33% Cu-doped TiO_2_ NPs increased both TAMs and TILs. Furthermore, analysis of the level of CD8^+^ TILs, revealed a significant increase in cytotoxic CD8^+^ T cells upon treatment with 33% Cu-doped TiO_2_ NPs (Fig. 4C). Analysis of the TME composition at earlier time points (2, 5, 8 days post NP administration) resulted in far less outspoken effects (data not shown), indicating a slow but gradual tumor-activating effect of the 33% Cu-doped TiO_2_ NPs. Upon looking at the 10% and 33% Cu-doped TiO_2_ NPs, both NPs consisted out of metallic Cu and the TiO_2_ matrix, suggesting that either one or both of these components plays a major role in TIL levels. The activation of TAMs by 33% Cu-doped TiO_2_ NPs and the associated anti-tumor immune response by cytotoxic CD8^+^ T cells, may be due to the presence of Cu^+^ in these samples, which was absent for the 10% Cu-doped TiO_2_ NPs.

A proper antitumor T cell response requires type I IFN-mediated activation of the Batf3-dependent CD103^+^ DC [32–34]. To gain more insight into the immune pathways involved in the antitumor responses mediated by 33% Cu-doped TiO_2_ NPs, the influx of DCs in the tumor draining lymph node was analyzed by image-based flow cytometry at 14 days post NP administration. Batf3-dependent DCs (conventional type 1 DCs or cDC1) and IRF4-dependent DCs (cDC2) represent the two major classes of DCs and can be discriminated by their distinct expression of XCR1 (cDC1) versus CD172α (cDC2) [35]. Compared to mock treated mice, a strong influx of cDC1 and cDC2 DCs was apparent in the draining lymph nodes in mice treated with 33% Cu-doped TiO_2_ NPs (Fig. 4D). As the XCR1^+^ DCs (cDC1) are linked to the anti-tumor activation of cytotoxic CD8^+^ T cells [36], this may explain the higher levels of CD8^+^ TILs in the treated tumor samples. Analysis of splenocytes obtained from tumor-bearing animals revealed only insignificant changes in the number of B or T cells or macrophages, nor was there any effect on the CD8/CD4 ratio or the T cell activation status (Fig. 4E–K). These data demonstrate a potent localized anti-tumor immunization, which remains limited in systemic immune responses under the conditions used. To confirm the latter finding, we grafted mice with two tumors, one on the right and a second on the left flank and treated all animals with anti-PD1 therapy but only treated the tumor on the right with 33% Cu-doped TiO_2_ NPs. This resulted in a reduced growth of both tumors compared to animals only receiving anti-PD1 antibodies, but this was significantly enhanced in the right tumor, compared to the left tumor (Fig. 4L), corroborating our findings that the observed immune response due to the 33% Cu-doped TiO_2_ NPs remains fairly localized.

### Mechanistic investigation of 33% Cu-doped NP therapeutic efficacy

**Fig. 5:**
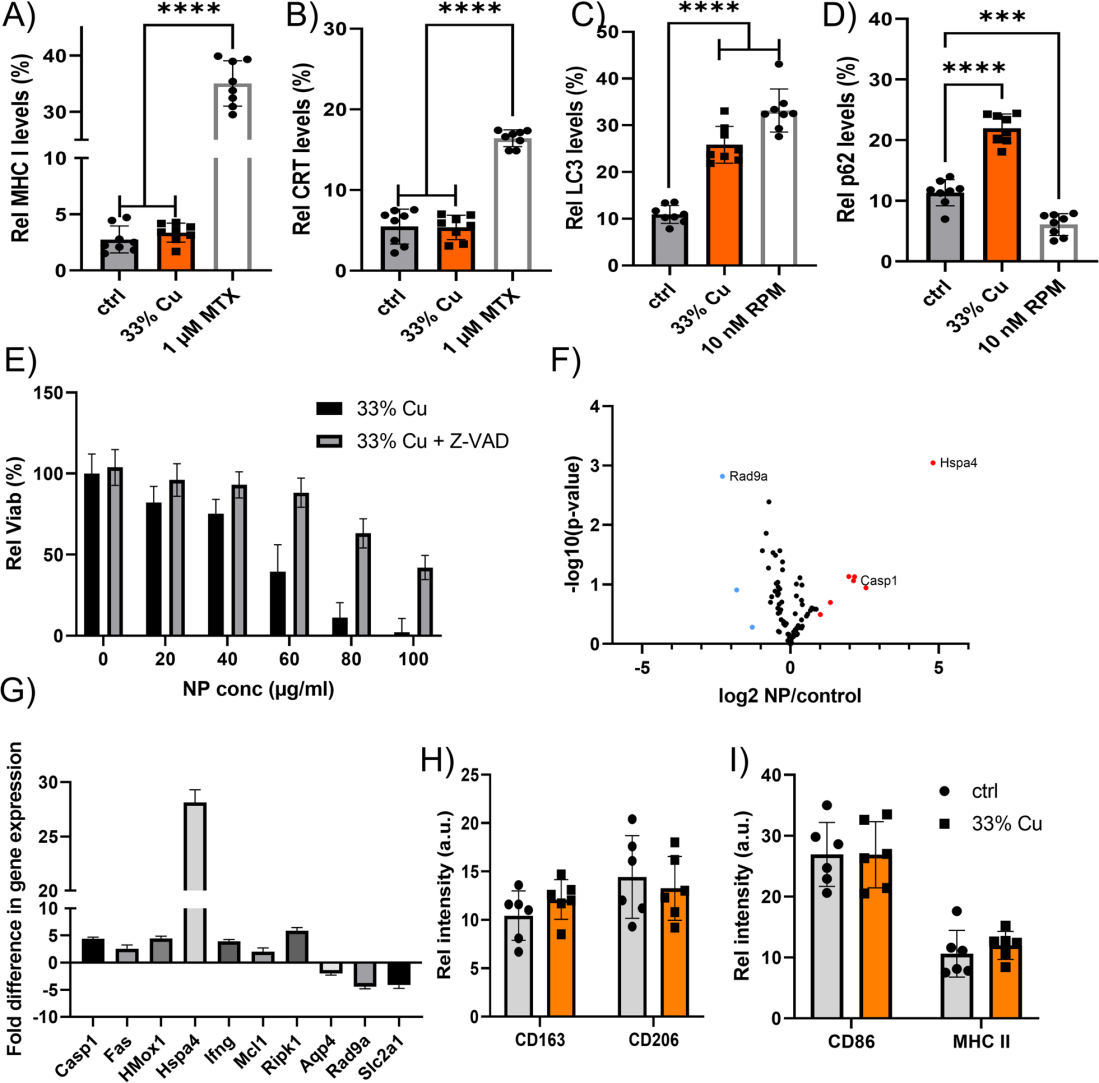
**A**, **B** Histograms displaying the relative level of A) surface-located MHC-I, B) surface-located calreticulin in KLN 205 cells exposed to saline control, 33% Cu-doped TiO_2_ NPs or 1 µM mitoxanthrone as positive control expressed as the relative level of cells positive for the marker out of the total population of cells analysed by ImageStreamX Mark II analysis. **C**, **D** Histograms displaying the relative level of C) autophagy marker LC3, D) autophagic flux marker p62 in KLN 205 cells exposed to saline control, 33% Cu-doped TiO_2_ NPs or 10 nM rapamycin as positive control expressed as the relative level of cells positive for the marker out of the total population of cells analysed by ImageStreamX Mark II analysis. **E** Histogram showing the viability of KLN 205 cells treated with 33% Cu-doped TiO_2_ NPs at the concentrations indicated in the presence and absence of caspase-inhibitor Z-VAD-fmk. Data are expressed as mean ± SEM (*n* = 4). **F** Volcano plot for 84 genes related to cellular stress and toxicity analyzed in untreated KLN 205 cells and 33% Cu-doped TiO_2_ NPs at 20 µg/ml for 24 h, where significant differences in expressed genes are indicated in orange (overexpression) or blue (underexpression). **G** The significant genes found in F) are further indicated with their respective fold difference compared to untreated KLN 205 cells. Data are expressed as mean ± SEM (*n* = 4). **H**, **I** Histograms of RAW 264.7 macrophages either untreated or exposed to 33% Cu-doped TiO2 NPs at 40 µg/ml for 24 h, displaying the level of surface-located H) M2 macrophage markers (CD163, CD206) or I) M1 macrophage markers (CD86, MHC-II). Significant differences between a treated group and untreated controls at the same time point are indicated where relevant (p < 0.001: ***; p < 0.0001: ****) based on ANOVA testing using GraphPad Prism 9 (*n* = 8)

The observed immunomodulation could be caused by a variety of possible mechanisms. As a first candidate, it was evaluated whether tumor cells exposed to the NPs would die in an immunogenic manner. For this, upregulation of calreticulin I and MHCII at the cell surface was investigated, being hallmarks of immunogenic cell death [37]. Figure **5**A, B reveal insignificant upregulation of either of the two markers during cell death, which suggests that the cells die in a non-immunogenic manner. Given the broad variety in possible cell death mechanisms, and looking at the mitochondrial effects observed in Fig. 2D cells were again exposed to 33% Cu-doped TiO_2_ NPs, and evaluated for LC3b clustering, a hallmark for cellular autophagy. Figure 5C reveals a clear increase in cellular autophagosome levels upon exposure of the cells to the 33% Cu-doped TiO_2_ NPs. As autophagosome accumulation can be linked to either the induction of autophagy, or impeding autophagosome-lysosome fusion, as described for Au NPs [38], this was further evaluated by studying the degradation of p62/SQSTM1, a protein known to be degraded specifically via the autophagy pathway [39]. Figure 5D demonstrates an increase in p62 levels, suggesting that the higher levels of autophagosomes is due to impeded lysosome-mediated turnover of autophagosomes. Interestingly, neither of these findings were observed for pure CuO NPs (data not shown), suggesting that the TiO_2_ matrix is likely the predominant cause of the observed effects. Previous reports have suggested that TiO_2_ NPs can interact with the lysosomal compartment and hereby induce autophagy [40].

As autophagy is mainly seen as a cytoprotective mechanism, which can turn cytotoxic upon excessive levels, the influence of lysosomal impairment on cell viability remains unclear. As both CuO and TiO_2_ NPs have also been frequently reported to induce apoptosis [41], cells were exposed to 33% Cu-doped TiO_2_ NPs in the presence of Z-VAD-fmk (a pan-caspase inhibitor), which to a large extent was able to prevent NP-induced cell death (Fig. 5E). These data indicate that cell death seems to be primarily caused by apoptosis, and that the contribution of autophagy in cell death is limited. This is in line with published data on both pure TiO_2_ NPs and CuO NPs, which have mainly been linked with apoptosis [41]. Gene expression analysis on KLN205 tumor cells exposed to 33% Cu-doped TiO_2_ NPs was then performed, focusing on cellular stress and toxicity pathways. In line with the data above, the significantly altered genes primarily indicated apoptosis as the key mechanism involved (Fig. 5F, G). One gene in particular, *Hsp4a* stood out as an interesting candidate for further investigation, due to the high upregulation and the known role of its protein (HSP70) as a danger-associated molecular pattern (DAMP) that can elicit immune responses [42].

As apoptosis is a non-immunogenic cell death mechanism, the immunomodulatory effects of the 33% Cu-doped TiO_2_ NPs may stem from other effects. One other mechanism lies in the polarization of TAMs, as has been described for iron oxide NPs [9]. However, interrogation of macrophages exposed to the NPs showed only an insignificant shift from immunosuppressive M2 TAMs into inflammatory M1 TAMs (Fig. 5H, I).

### Effects of 33% Cu-doped NP on dendritic cells

**Fig. 6:**
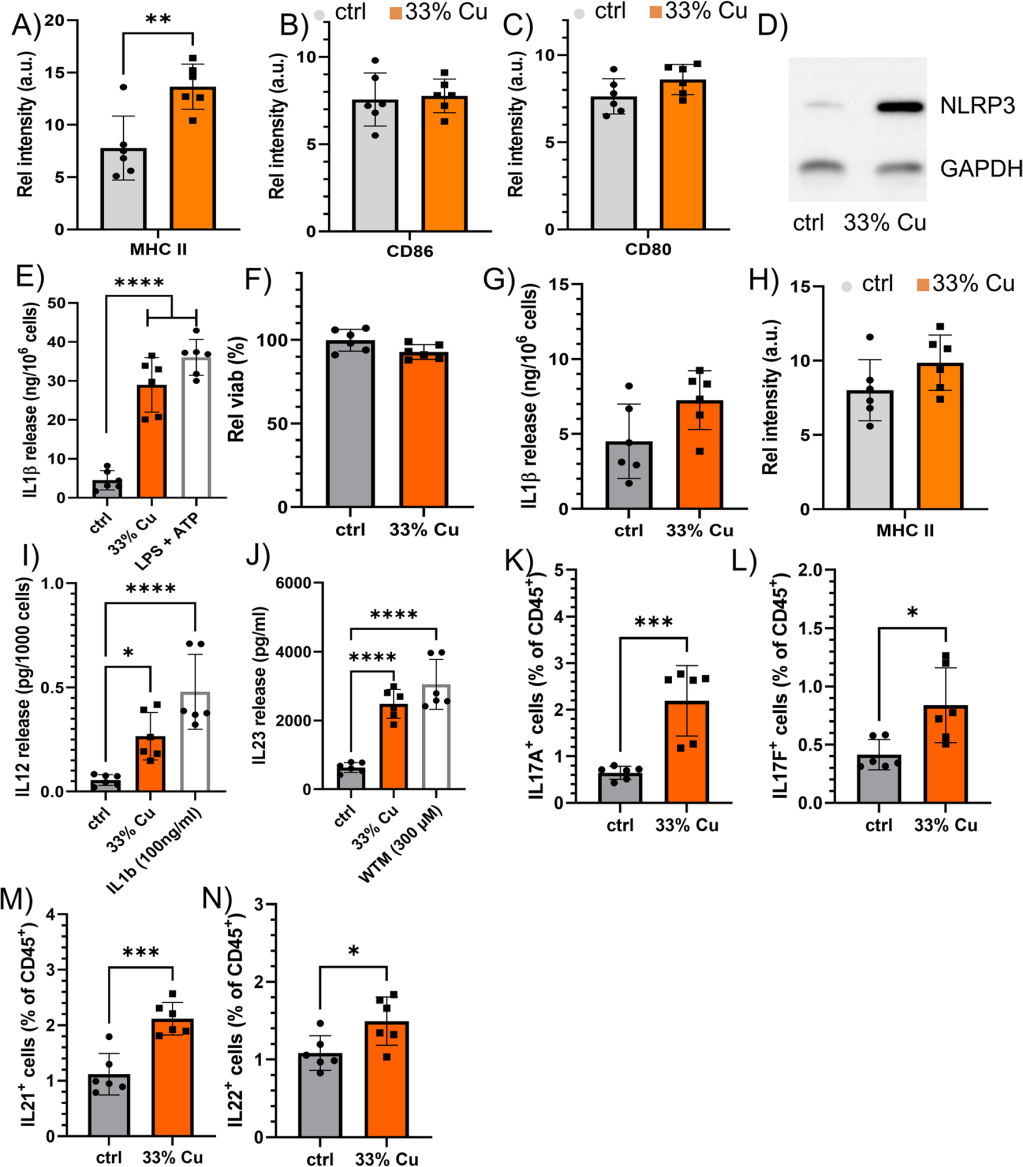
**A**–**C** Histograms displaying the relative level of A) MHC-II, B) CD86, C) CD80 in in vitro cultured DCs exposed to saline (control) or 33% Cu-doped TiO_2_ NPs. **D** Western blot of in vitro cultured DCs exposed to saline (control) or 33% Cu-doped TiO_2_ NPs and analyzed for the expression level of NLRP3 and GAPDH as a housekeeping control. **E** Histogram showing the amount of IL1β released by in vitro cultured DCs either exposed to saline (control), 33% Cu-doped TiO_2_ NPs or a positive control of LPS + ATP. **F** Histogram displaying the viability of in vitro cultured DCs exposed to 33% Cu-doped TiO_2_ expressed relative to the viability of untreated DCs. **G**, **H** Histogram showing G) the amount of IL1β released or H) MHC-II levels by in vitro cultured DCs either exposed to saline (control) or 33% Cu-doped TiO_2_ NPs in the presence of NLRP3-inhibitor MCC950. **I**, **J** Histograms indicating the level of I) IL12 and J) IL23 secreted by in vitro cultured DCs exposed to saline (vehicle control), 33% Cu-doped TiO_2_ NPs or positive controls (100 ng/ml IL1β for I) and 300 µM wortmanin for J)). **K–N** Histograms indicating intracellular cytokine levels indicative of Th17 cell types obtained from tumor samples either treated with vehicle control (saline) or 33% Cu-doped TiO_2_ NPs for K) IL17A, L) IL17F, M) IL21, N) IL22. These data are expressed as the level of cytokine-positive lymphocytes relative to the total number of lymphocytes. Significant differences between a treated group and untreated controls at the same time point are indicated where relevant (p < 0.05: *, p < 0.01: **; p < 0.001: ***; p < 0.0001: ****) based on ANOVA testing using GraphPad Prism 9 (*n* = 6)

We next looked into the effect of the 33% Cu-doped NP on dendritic cells. For the in vitro studies, we used a conditionally immortalized DC cell line, which was described before [43]. It is important to note that for in vitro assays, expression of the immortalization gene was switched off (please see Materials and Methods Sect. "De-immortalization of DCs" for technical details), and cells had been found to behave similar to freshly isolated DCs [43]. While the effect of the immortalization process is therefore expected to be minimal, it cannot be ruled out completely. Exposure of DCs to the 33% Cu-doped TiO_2_ NPs resulted in higher levels of MHCII, as was also observed for DCs in the tumor-draining lymph nodes, insignificantly affected activation markers CD80 or CD86 (Fig. 6A–C). Western blotting revealed activation of NLRP3, a key mediator in inflammasome activation and pyroptosis, but while ELISAs did detect a significant increase in IL1β release (Fig. 6D, E), viability remained unaffected (while loss of viability is typical for pyroptosis) (Fig. 6F). As IL1β is an important mediator in generating CD8^+^ T cells [44], and could thus explain the increase in local CD8^+^ TILs in the tumor, DCs can also induce other lymphocytes [45]. Hyperactivation of DCs has been reported, where DCs have been found to be triggered into IL1β secretion for longer time periods, without any loss of cell viability [46]. The hyperactivated state prevents the DCs from pyroptosis and is characterized by the predominant differentiation of Th1/Th17 CD4^+^ T cells [47]. This was confirmed by exposing cultured DCs to the NPs, in the presence of MCC950, a known inhibitor of NLRP3, which prevented IL1β secretion and abrogated the NP-induced MHCII activation (Fig. 6G, H).

DCs exposed to 33% Cu-doped TiO_2_ NPs were also found to secrete higher levels of IL12 and IL23, which are Th1/Th17 polarizing cytokines (Fig. 6I J) [48]. To confirm whether Th17 cells played a vital role in the therapeutic effect of the 33% Cu-doped TiO_2_ NPs, tumors having received the 33% Cu-doped TiO_2_ NPs were analyzed and compared for the expression of Th17-secreted factors. Figure 6K–N reveal significant elevation in IL-17A, IL-17F, IL-21, and IL-22, which are specific for Th17 cells and thus confirm the presence of Th17 cells induced by hyperactivated DCs due to the NPs.

### A shortlived hyperactivation of DCs drives local anti-tumor immune response

**Fig. 7:**
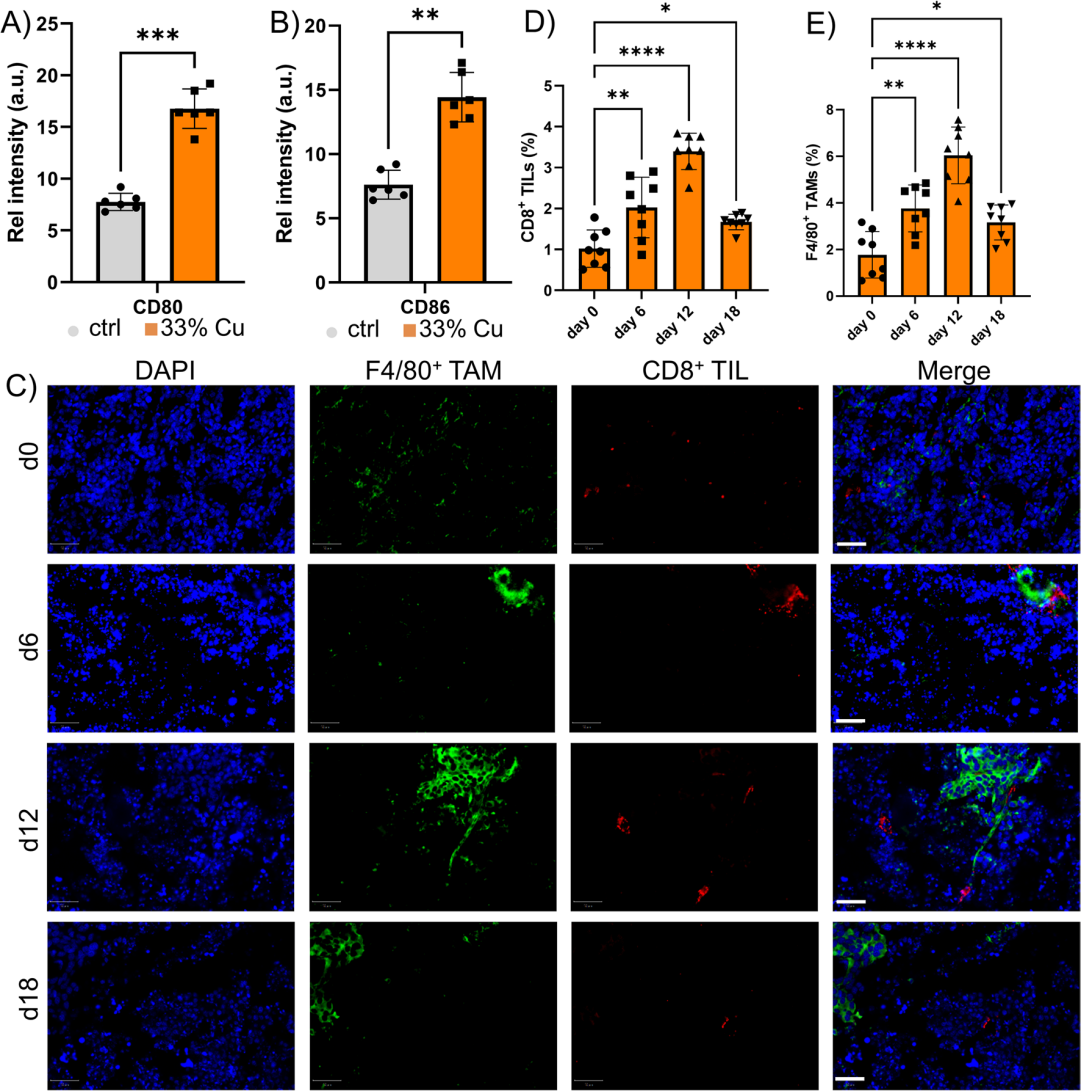
**A**, **B** Histograms displaying the relative level of A) CD80 and B) CD86 in in vitro cultured conditionally immortalized DCs exposed to supernatant of E0771 cells that were previously incubated with either saline (control) or 33% Cu-doped TiO_2_ NPs. **C** Representative micrographs of tumor sections obtained from animals bearing KLN 205 subcutaneous tumors treated with 33% Cu-doped TiO_2_ NPs. Images are shown of tumors isolated 0, 6, 12 or 18 days following NP administration. The tissue sections were counterstained with DAPI nuclear stain (blue), following staining against F4/80 (green, TAMs) and CD8 (red, cytotoxic T cells). The images on the right show the merged images. Scale bars: 250 µm. **D**, **E** Histograms indicating the level of D) CD8^+^ TIL and E) F4/80^+^ TAMs expressed relative to the total number of cells in the tumor section as analyzed using QuPath analyzer for tissues obtained 0, 6, 12 or 18 days following NP administration

As demonstrated by the CD8^+^ TIL increase and the inhibitory effect of anti-CD8 antibodies, the antitumor effect was however mainly driven by CD8^+^ T cells. Here, we looked into the possible role of the strongly upregulated *Hspa4* gene. Heat shock proteins (HSP) are known to have a complex role in immunomodulation, as they are able to interact directly with different Toll-like receptors and are capable of promoting antigen presentation of chaperoned peptides through interactions with receptors on DCs [49]. When tumor cells were exposed to the 33% Cu-doped TiO_2_ NPs, the supernatant resulted in higher levels of HSP, released by dying cells. When DCs were exposed to this supernatant, it resulted in a potent activation of the DCs, as observed by CD80 and CD86 upregulation (Fig. 7A, B). These data suggest a more pronounced CD8^+^ T cell activation, and could result in an increase in T cell influx into the tumor. To evaluate this, IHC was performed of animals bearing KLN-205 tumors, where samples were analyzed at different time points depending on the 33% Cu-doped TiO_2_ NP treatment strategy. A major influx of both CD8^+^ T cells as well as F4/80^+^ TAMs was observed at 6 days post treatment, but this decreased significantly by 12 days and remained at low, but slightly elevated levels compared to pretreatment conditions (Fig. 7C–E). The transient nature of the immune response was striking, and we studied the behavior of the different cell components known to be involved. For the DCs, exposure to the 33% Cu-doped TiO_2_ NPs resulted in a gradual, but prolonged release of IL1β, which reached a maximum around 3 days post exposure, and then decreased with time. For the cancer cells exposed to the NPs, HSP release in the supernatant was maximal after 24 h and decreased gradually until near normal levels by 4 days post exposure. The differences in kinetics of maximal activation could explain the transient effects observed. Where tumor-associated antigens may be shuttled out of the cells as they are dying due to HSPs that can then be taken up by DCs, this happens at an early stage, prior to DCs reaching full activation status yet. At this stage, some DCs may get triggered into promoting a CD8^+^ T cell response, but with the lack of any strong novel antigen (the KLN205 tumors are rather poorly immunogenic), a weak specific tumor response could be triggered that would further lead to either a systemic immunization or the generation of memory T cells. The activation of the DCs by the NPs themselves would then promote the Th17 response, but with the lack of proper tumor antigens present, this seems to limit tumor-specific CD8^+^ TIL proliferation and activation.

### Cu-doped TiO_2_ NPs enhance dendritic cell vaccines

**Fig. 8:**
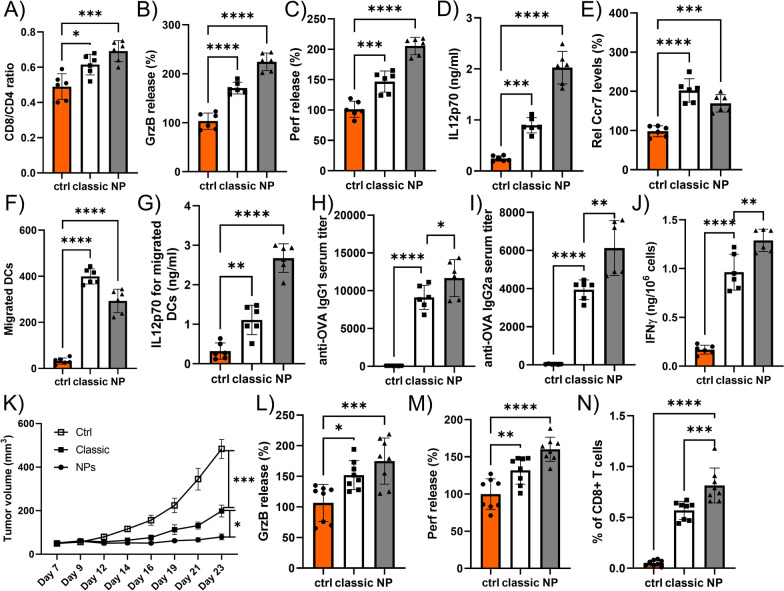
**A** Histogram displaying the ratio of CD8^+^ T cells over CD4^+^ T cells obtained upon in vitro stimulation by OVA-peptide loaded DCs that had either been left untreated (control) or were activated classically, or upon exposure to 33% Cu-doped TiO_2_ NPs. **B**, **C** Histograms displaying the relative level (control: 100%) of B) granzyme B release, or C) perforin release from T cells obtained from part A that had been exposed to the OVA-derived SIINFEKL peptide. **D**, **E** Histograms displaying the level of D) IL12p70 release or E) relative Ccr7 surface expression (control: 100%) on DCs that had either been left untreated (control) or were activated classically, or exposed to 33% Cu-doped TiO_2_ NPs. **F**, **G)**Histograms displaying the F) number of migrated DCs or G) the level of IL12p70 secreted from migratory DCs obtained from DCs that had either been left untreated (control) or were activated classically, or exposed to 33% Cu-doped TiO_2_ NPs and subsequently exposed to the CCR7 ligand 6C-kine. **H**, **I** Histograms displaying the in vivo antibody titer for H) OVA-specific IgG1 or I) OVA-specific IgG2α obtained upon intravenous administration of OVA-pulsed DCs that had either been left untreated (control) or were activated classically, or exposed to 33% Cu-doped TiO_2_ NPs. **J** Histogram displaying the level of IFNγ obtained from ex vivo splenocytes obtained from animals receiving the different types of OVA-pulsed DCs and incubated with OVA ex vivo. **K** Tumor volumes obtained from OVA-expressing E0771 tumors grafted subcutaneously in C57Bl6 mice receiving the different OVA-pulsed DCs that had either been left untreated (control) or were activated classically, or exposed to 33% Cu-doped TiO_2_ NPs. **L**, **M** Histograms displaying the relative level (control: 100%) of L) granzyme B release, or M) perforin release from CD8^+^ T cells isolated from OVA-E0771 tumor-bearing animals having received OVA-pulsed DC grafts of the different DC types and then exposed to the OVA-derived SIINFEKL peptide ex vivo. **N** Histogram displaying the level of OVA-specific CD8^+^ T cells isolated from the spleen of OVA-E0771 tumor-bearing animals having received OVA-pulsed DC grafts of the different DC types

While the observed localized and transient effects of the 33% Cu-doped TiO_2_ NPs reduce the suitability for long-term systemic immunization strategies in the manner in which they were used now, the observed effects demonstrate a strong potential for these NPs to serve as agent in DC anti-tumor vaccination strategies. Thus far, DC vaccination has remained troublesome, mainly due to the difficulty in priming and activating the DCs in the correct manner to enable a proper anti-tumor response. A classical golden standard for generation of DC vaccines is the use of a cytokine cocktail, based on tumor necrosis factor α (TNF α)/interleukin 1β (IL-1 β)/interferon γ (IFNγ)] [50]. An effective DC vaccine should consist of fully mature DCs that express high levels of costimulatory molecules and that efficiently migrate to the lymph nodes upon Ccr7 stimulation. Enhanced levels of tumor-specific Th1 cells and cytotoxic T lymphocytes, enabling augmented tumor rejection has been linked with high secretion of interleukin-12p70 (IL-12p70). To date, various cytokine cocktails have been evaluated, without reaching the ideal conditions required to generate excellent DC vaccines. Given the clear activation effect of the 33% Cu-doped TiO_2_ NPs on DCs, we then evaluated whether these NPs could contribute to a better DC vaccination strategy compared to wild-type and classically activated (TNFα/IL-1β/IFNγ) DCs. As a first test, DCs were first activated by either the NPs or activation cocktail, and after 2 days, they were pulsed with ovalbumin as a powerful antigen. DCs were then incubated with primary T cells, which resulted in a high increase of CD8^+^ T cells over CD4^+^ T cells compared to wild-type and classically activated DCs (Fig. 8A). When the T cells were incubated with the SIINFEKL peptide, their activity levels were far higher, as demonstrated by the significant increase in secreted granzyme B and perforin levels (Fig. 8B, C). Furthermore, the DCs revealed significantly higher levels of IL-12p70 upon NP stimulation (Fig. 8D), indicative of the strong induction of cytotoxic T lymphocytes. While Ccr7 expression levels in the DCs were significantly higher than for wild-type DCs, they were lower than for classically activated DCs (Fig. 8E), which also translated itself into reduced migratory capacity of the DCs in response to the CCR7 ligand 6C-kine (Fig. 8F). However, when analyzing the IL-12p70 secretion levels of migrated DCs, NP-stimulated DCs by far outperformed classically activated DCs (Fig. 8G), indicating that the reduced migratory capacity was more than compensated for by the enhanced induction capacity for cytotoxic T lymphocytes.

To evaluate the potential therapeutic potency of the NP-treated DCs, we explored the ability of the OVA-pulsed DCs to induce antigen responses against OVA. Figure 8 H and I indicate that both classically activated as well as NP activated DCs resulted in Th1 and Th2 responses, as demonstrated by the IgG1 and IgG2α responses, respectively. Both Th1 and Th2 responses were, however, stronger for the NP-stimulated DCs than for the classically activated ones, which is in line with the in vitro data demonstrating a higher activation of T cell responses. Furthermore, when spleen cell suspensions from these mice were activated ex vivo with OVA and the IFN-γ production was measured, we found that NP-stimulated DCs enhanced the OVA-specific IFN-γ responses compared to classically activated DCs (Fig. 8J).

In a final test, DCs were administered intravenously in C57Bl6 mice grafted with a subcutaneous OVA^+^ breast adenocarcinoma (E0771) tumor. Wild-type DCs were taken as a control condition, which resulted in a rapidly growing tumor (Fig. 8K). Administration of classically activated DCs significantly delayed rapid tumor growth, but these effects were further improved upon using NP-stimulated DCs. These results show irrespective of the lower Ccr7 expression for NP-stimulated DCs and the somewhat lower in vitro migratory capacity of the DCs, the enhanced activation of cytotoxic T lymphocytes result in a significant increase in therapeutic efficacy. These findings were further supported by ex vivo analysis of isolated CD8^+^ T cells from the tumor, which revealed higher levels of granzyme B and perforin release (Fig. 8L, M) upon exposure to SIINFEKL peptide, while isolated splenic CD8^+^ T cells reached significantly higher levels (Fig. 8N).

## Discussion

The 33% Cu-doped TiO_2_ nanoparticles were found to obtain transient and localized anti-tumor immune responses, even in the absence of potent tumor antigens. A mechanistic investigation revealed indirect cancer-selective toxicity for any of the formulations. While some cell type-dependent variability is apparent, the non-cancerous cell types display an equal sensitivity towards the NP-induced toxicity. This is in contrast with some other formulations, such as ZnO or CuO NPs, where higher toxicity levels have been associated with cancerous cell types [12, 31]. It has been suggested that this could be explained due to the natural lower pH surrounding tumors, which would promote NP dissolution even when the NPs are not internalized by the cancer cells yet. However, for in vitro cultures, using buffered media, the influence of pH changes among the cell types will be negligible. Another potential mechanism for cancer cell-selective toxicity has been linked with the ROS generated by these NPs. For most cells, ROS is often generated in mitochondria as a byproduct of the oxidative phosphorylation process. In cancer cells, the required energy levels are higher and this thus results in higher ROS production in mitochondria [51]. When additional ROS is then induced by engineered NPs, this may exceed an internal threshold for the cell, and hereby cause toxicity. As normal, non-cancerous cells tend to divide slower and are metabolically less active, this may prevent excessive ROS generation and avoid reaching the toxicity threshold [52]. The reduced proliferation rate of normal cells can also impact NP endocytosis levels, as the level of NP uptake has been shown to be strongly associated with cell cycle progression [53]. This would furthermore result in lower numbers of internalized NPs, which in turn would reduce NP-mediated toxicity.

The lack of a systemic effect and the observation of a mere transient therapeutic response is likely due to the lack of a proper tumor-specific antigen which could be processed by the DCs. Syngeneic murine models, like the KLN205 tumors in DBA2 mice, are generally poorly immunogenic and thus suppress any proper immune response, even upon use of clinically approved immune checkpoint inhibitors [54]. This low immunogenicity of syngeneic murine models is apparent in the composition of the tumor microenvironment (TME), which is very low in TAMs and TILs, and mainly consists of tumor cells themselves. This is in stark contrast with many human tumor models, where the TME can easily consist out of more TAMs and TILs than actual tumor cells [55]. In many studies focusing on immunotherapy in syngeneic murine models, a strong model antigen (typically chicken ovalbumin) is used, in order to enhance the immunogenicity of the tumors cells and to ensure a potent and unique antigen being released upon immunogenic cell death. While the observations in our study seemed to have rather poor therapeutic outcomes at first glance, it is therefore important to take into consideration the lack of any model antigen being used, and the nice therapeutic effect that could be observed for the NPs when used together with anti-PD1 antibodies, while the immunotherapy as a monotherapy lacked any significant improvement in tumor treatment. The Cu-doped TiO_2_ NPs are therefore potentially more potent in human tumors than in the murine models used here. In terms of clinical translation, one important aspect to take into account is the fact that the NPs generated here are ‘bare’, in the sense that they were not coated with any polymeric or lipid-based shell to provide colloidal stability in a physiological environment. Therefore, for the therapeutic in vivo studies, the NPs were administered locally at the tumor site and not systemically. The local administration of the NPs may prove difficult in some situations, but local administrations are gaining more interest in clinical settings as well [56]. This is mainly relevant in immunotherapy-based studies, where, if a potent immune response can be triggered against a primary tumor, it can be expected that local or even distant metastases will also be targeted by the activated immune system of the patient. As such, even upon local treatment, the final therapeutic response can very well be systemic. It would also be an option to chemically modify the surface of the NPs and provide a suitable biocompatible coating. However, it is important that this coating does not impede copper ion release kinetics, nor affects the ability of the TiO_2_ matrix to activate inflammasomes. Upon systemic administration, biodistribution would also need to be carefully monitored to know whether sufficient NPs reach the tumor, and whether accumulation of the NPs in other organs may elicit any unwanted toxicity. In this study, upon local administration, the majority of NPs were found to still be present in the tumor after several weeks, with only minimal amounts ending up in the liver and spleen (data not shown), while for systemically administered NPs, the distribution will be very different.

The different kinetics of cell activation are also linked to the mechanisms behind the observed cellular effects. The 33% Cu-doped TiO_2_ result in rapid cancer cell death, similar to free CuO NPs. In view of the different observations made between NP types and cell types, the potent therapeutic potential of the 33% Cu-doped TiO_2_ NPs is likely due to the occurrence of both metallic Cu and reduced Cu^+^ ions, as this combination is missing in the other formulations. While Cu-based NPs are generally known to induce apoptosis, as was also observed in our study, Cu^2+^ and Cu^1+^ ions have been successfully used to stimulate immunogenic cell death [30, 57]. While classical ICD markers only showed marginal increases, the 33% Cu-doped TiO_2_ NPs did elicit the release of HSP70, a known DAMP and in doing so, it can trigger an anticancer response specific towards tumor cells that have died upon ingesting the NPs. This would support our previous observation where 33% Cu-doped TiO_2_ NPs resulted in elevated cytotoxic CD8^+^ T cells, while this was absent for 10% Cu-doped TiO_2_ NPs. The enhanced activation of DCs, essential for driving the TIL influx and activation, is then likely the result of the metallic Cu in combination with the TiO_2_ matrix, as TiO_2_ and other metallic NPs such as gold, silver as well as Cu have all been found to be potent inducers of inflammation [58–61]. These observations are mainly linked to the triggering of inflammasome NLRP3, as has been reported in literature [62]. As previously mentioned, other Cu-based NPs have already been described, such as copper-cysteamine NPs [17, 18]. These, and other agents, have been found to succesfully induce oxidative stress which in turn have been found to trigger anticancer immunity [16]. We believe that the combination of copper ions in a TiO_2_ matrix acts in a dual manner, where the copper ions can induce immunogenic cell death and act directly on the tumor cells, while the TiO_2_ matrix will act as an adjuvant to boost the strength of this immune response. This is similar to the role of aluminum-based salts, used for decades in multiple vaccine formulations [63]. The crystal structure of NPs is a known trigger for NLRP3 inflammasomes. When taken up by TAMs, this will trigger recruitment of innate immune cells such as neutrophils and subsequently dendritic cells to the tumor site. The latter will, in turn, activate the adaptive immune system. The main role of the TiO_2_ matrix therefore is dual, where it serves as a reservoir for the controlled release of copper ions to generate oxidative stress at the lysosomal level, while it also acts as a direct activator of NLRP3 inflammasomes and hereby functions as an adjuvant, where it boosts the immune response, which is of particular interest in the case of cancer immunotherapy, where strong and potent antigens are often lacking.

In the end, the nanoparticles enable finely tuned generation of dendritic cells that surpass classically activated dendritic cells in therapeutic efficacy against solid tumors. For this, dendritic cells can be treated with the NPs and pulsed with tumor antigens, resulting in highly efficient therapeutic efficacy that promises to have a profound impact on human health.

## Materials and methods

### NP synthesis and characterization

Flame spray pyrolysis was utilized for the production of pure TiO_2_, pure CuO and Cu doped TiO_2_, nanoparticles. Titanium (IV) isopropoxide (TTIP, Aldrich, purity 97%) and copper (II)-naphthenate dissolved in xylene (Strem Chemicals, 99.95% pure, with total metal concentration of 0.5 M) were precursor-solvent combinations for the synthesis of pure TiO_2_ and CuO. To prepare 1%, 5%, 10%, 33% Cu doped TiO_2_ nanoparticles, 49.5 mL, 47.5 mL, 45 mL, 33.5 mL titanium (IV) isopropoxide precursor solution (0.5 M Ti) was mixed with 0.5 mL, 2.5 mL, 5 mL, 16.5 mL copper (II)-naphthenate solution (0.5 M Cu) before combustion, respectively. While premixed gases flow 1.5L/min CH_4_ and 3.2L/min O_2_ were used to form a spray flame, 5 mL/ min of the liquid precursor solutions were delivered at the flame nozzle and atomized using 5L/min O_2_ at 1.5 bar pressure at the nozzle tip [64–66]. Through nucleation, surface growth, coagulation and coalescence, ultrafine particles were formed and collected from 257 mm glass filters placed at distance of 60 cm from the nozzle.

For characterization, the collected NPs were analyzed by XRD, TEM and EELS. X-ray powder-diffraction data for all samples were collected on a Bruker D8 Discover equipped with a primary Johansson monochromator producing Ni filtered Cu Kα (λ = 0.154 nm) radiation. Continuous scans in the range of 10 90° 2θ were applied with an integration step width of ~ 0.03° 2θ and 30 s per step. The data obtained from XRD measurements were refined with a Rietveld refinement using BRASS program. Scale factor, unit cell parameters, background, sample displacement, Gaussian and Lorentzian peak width parameters were refined step by step. Anatase (ICSD 172,916), rutile (ICSD 9161) and CuO (ICSD 69,757) were used as structure models. The instrumental contribution to the peak broadening was taken into account using instrumental parameters derived from standard crystalline LaB_6_. The quality of refinement was evaluated by R_wp_ and residual Bragg factor R_Bragg_. Phase compositions, average crystallite size (d_XRD_) and densities of each sample were determined.

For TEM measurements, a small portion of Cu-doped TiO_2_ NPs (~ 1–2 mg) was dispersed in 5 mL of ethanol (AR grade, Strem) in an ultrasonic bath and sonicated for 15 min. A drop of the sample solution was placed on a carbon-coated copper grid. The samples were dried at ambient conditions and loaded in a FEI Titan 80/300 microscope equipped with a Cs corrector for the objective lens, a Fischione high angle annular dark field detector (HAADF), GATAN post-column imaging filter and a cold field emission gun operated at 300 kV acceleration voltage.

In order to figure out the oxidation state of Cu in the Cu doped TiO_2_, EELS was conducted on 10% and 33% Cu doped TiO_2_. The GATAN parallel EELS spectrometer was operated at 0.2 eV per channel as an energy dispersive detector. Spectra were taken at each sampling point in order to identify the homogeneity of the samples. By measuring the energy loss from the transmitted beam, the information around the central atoms is derived. EELS is then applied for determination of oxidation states of the transition metals. Core loss edge reflects the elemental composition. Near edge fine structure (ELNES) shows bonding and oxidation state. Distribution of near neighboring atoms is exhibited in extended energy loss fine structure (ExELFS).

### Cell culture

The following cell types were used in this study: human non-small lung adenocarcinoma (A549), human bronchial epithelial cells (Beas-2B), murine lung squamous tumor cells (KLN 205), human cervical cancer cells (HeLa) and murine breast adenocarinoma (E0771). A549, Beas-2B, HeLa and E0771 cells were cultured in high glucose containing Dulbecco's modified Eagle's medium (DMEM), supplemented with 10% fetal calf serum, 1 mM sodium pyruvate, 2 mM L-glutamine, and 1% penicillin/streptomycin (Gibco, Invitrogen, Belgium). KLN 205 cells were cultured in high glucose DMEM, supplemented with 10% fetal calf serum, 1 mM sodium pyruvate, 2 mM L-glutamine, 1% nonessential amino acids and 1% penicillin/streptomycin. All cell types were maintained in a humidified atmosphere at 37 °C and 5% CO_2_ and split 1/5 upon reaching 80% confluency.

For studies with DCs, conditionally immortalized DCs were used, as initially described in Richter et al. [43]. For culture, the DCs were kept in their immortalized form (large T antigen expression) in complete RPMI medium supplemented with 10% FBS, 2 mM L-glutamine, 100 IU/ml penicillin, 100 µg/ml streptomycin, 1 mM sodium pyruvate, 10 mM HEPES (Gibco), 50 µM β-mercaptoethanol (Sigma Aldrich) and GM-CSF (10 ng/ml), dexamethasone (Dex; 100 nM) and doxycycline (Dox; 1 µg/ml).

### Cell-nanoparticle interaction studies

For high-content imaging studies, all cell types were seeded at 3000 cells/well in a 96 well plate (Nunc, Belgium) after which the cells were allowed to attach overnight in a humidified atmosphere at 37 °C and 5% CO_2_. Subsequently, cells were incubated with the different Cu-doped TiO_2_ NPs for 24 h in full growth medium at concentrations of 0, 20, 40, 60, 80 and 100 µg/ml. Every condition was performed in triplicate and results were analyzed based on the three repeats. The high-content imaging experiments were performed based on previously validated methods. Experimental details are given in the following sections:

#### Cell viability and mitochondrial health

Following cellular exposure to (Cu-doped) TiO_2_ NPs, cells were washed twice with phosphate buffered saline (PBS; Gibco, Invitrogen, Belgium) and treated with 200 nM MitoTracker Red CMXRos and 2 µM fixable LiveDead Green dead cell stain (Molecular Probes, Life Technologies Europe, BV, Belgium) in 100 µl/well of PBS (with Ca^2+^ and Mg^2+^) and incubated in the dark for 30 min at room temperature. Next, the staining media was aspirated, cells were washed gently with PBS (3x) fixed with 4% paraformaldehyde (PFA) for 15 min at room temperature. The fixative was aspirated and cells were washed three times with PBS. Cells were then counterstained using Hoechst 33,342 Nuclear stain (20 µg/ml PBS in 100 µl/well) for 15 min at ambient temperature in the dark. The nuclear counterstain was then removed, cells were washed three times with PBS and 100 µl of PBS was added to every well, after which the plates were analyzed using the InCell 2000 analyzer (GE Healthcare Life Sciences, Belgium). During acquisition, a minimum of 5000 cells/condition were acquired (over 3 wells) using a 20 × objective for the following channels: UV/blue for Hoechst nuclear stain, FITC/FITC for the Live-Dead Green dead cell stain and DsRed/DsRed for the MitoTracker Red CMXRos stain. Data analysis was then performed on the InCell Investigator software (GE Healthcare Life Sciences, Belgium) using in-house developed protocols. The level of cell viability was calculated by segmenting cells based on the Hoechst stain and determining the perinuclear region by enlarging the nuclear stain 2.5-fold and using the original Hoechst stain images as seed images. Cell viability was then calculated by determining the number of total cells minus the number of dead cells (dead cells are defined as cells with clear green nuclei, where the intensity is minimally threefold above noise level and an area of minimally 2 µm^2^). These values were then normalized to control values (100%).

#### Mitochondrial health and ROS were calculated as follows

The DsRed/DsRed channel was segmented, using the nuclear target channel as seed images. Based on the segmented mitochondrial images, the overall area of cellular mitochondria were calculated, for any dot in the mitochondrial channel that had an intensity of minimum threefold higher than the background noise level. The total area of cellular mitochondria was determined as a marker for mitochondrial stress, where damaged mitochondria change shape, turning from an elongated to a more spherical morphology. The total area of cellular mitochondria was then normalized to the area of mitochondria in untreated control cells (100%). For mitochondrial ROS, the level of fluorescence intensity of the segmented mitochondria was determined. The intensity of the mitochondrial signal was then normalized to the intensity level of untreated control cells (100%).

#### Cell area and cell aspect ratio

After cellular exposure to (Fe-doped) CuO NPs, cells were washed (3x) with 500 µl PBS/well and fixed for 15 min at room temperature with 4% PFA. The fixative was then aspirated, cells were washed (3x) with PBS (500 µl/well) after which cells were permeabilised with 250 µl/well of Triton X-100 (1%) for 10 min at room temperature. Cells were then blocked with 10% serum-containing PBS for 30 min at room temperature. Next, cells were stained using 100 µl of staining solution per well of Acti-Stain 488 (Tebu-Bio, Belgium) and incubated for 90 min in the dark at room temperature. The staining solution was aspirated, cells were washed (3x) with PBS (100 µl/well) after which 100 µl fresh PBS was added to each well and the plates were kept at 4 °C in a dark container until analyzed using the InCell 2000 high-content imaging system. For acquisition, the following channels were selected: UV/blue for Hoechst nuclear stain, and FITC/FITC for the actin stain. Data analysis was then performed on the InCell Investigator software (GE Healthcare Life Sciences, Belgium) using in-house developed protocols, using a minimum of 5000 cells/condition. The size of the cells was calculated as follows: First, cell nuclei were segmented based on the blue channel. Cells were then segmented using the FITC channel, where any holes in the cells were filled up and included. Cells on the border of the field of view were excluded from the analysis. The segmentation was based on the blue channel as seed channel for the nucleus. The total area of every individual cell was then determined. Furthermore, to calculate the aspect ratio, the length of the major axis was determined, as well as maximum value for the minor axis (perpendicular to the major axis). The ratio of the minor axis over the major axis determines the aspect ratio.

### Analysis of immunogenic cell death

KLN205 cells were seeded at 1*10^5^ cells/flask in 25 cm^2^ cell culture flasks and allowed to settle overnight in a humidified atmosphere at 37 °C and 5% CO_2_. Media were then removed and fresh media (5 ml) was given with 33% Cu-doped TiO_2_ NPs at 40 µg/ml for 24 h. As a positive control, cells were treated with 1 µM of mitoxanthrone, a known inducer of immunogenic cell death. Media were removed, cells were washed with PBS, trypsinized and all supernatants, washing media and cells were added together and centrifuged at 2500 rpm for 6 min after which the cells were split into 2 fractions and one half was stained with anti-calreticulin antibody (AF647-anti-CRT clone EPR3924, Abcam) and the other half was stained with anti-MHC I antibody (FITC-anti-MHC-I clone 34–1-2S, Thermo Fisher Scientific) for 30 min at 4 °C. The cells were then centrifuged again, and resuspended in PBS after which they were run through the ImageStream X Mark II (Merck, Belgium) for analysis. Using iDEAS software, focused and single cells were selected and analysed for surface-localized calreticulin and MHC I levels.

### Analysis of autophagy

KLN205 cells were seeded at 1*10^5^ cells/flask in 25 cm^2^ cell culture flasks and allowed to settle overnight in a humidified atmosphere at 37 °C and 5% CO_2_. Media were then removed and fresh media (5 ml) was given with 33% Cu-doped TiO_2_ NPs at 40 µg/ml for 24 h. As a positive control, cells were treated with 10 nM of rapamycin, a known autophagy inducer. Media were removed, cells were washed with PBS, trypsinized and all supernatants, washing media and cells were added together and centrifuged at 2500 rpm for 6 min after which the cells were fixed (2% PFA), permeabilized (0.1% Triton X100) and then washed, centrifuged at 2500 rpm for 6 min after which the cells were split into 2 fractions and one half was stained with anti-LC3b antibody (DyLight650-LC3b clone PA5-22,937, Thermo Fisher Scientific) and the other half was stained with primary anti-p62 antibody (anti-p62/SQSTM1 clone MA5-32,835) followed by secondary AF488-goat-anti-rabbit antibody for 30 min at 4 °C. The cells were then centrifuged again, and resuspended in PBS after which they were run through the ImageStream X Mark II (Merck, Belgium) for analysis. Using iDEAS software, focused and single cells were selected and analysed for spot counting of intracellular LC3b or p62 foci.

### Analysis of cell death pathway

Cell death was investigated similarly as described above using a high-content imaging approach. In short, KLN205 cells were exposed to 33% Cu-doped TiO_2_ NPs for 24 h at 0, 20, 40, 60, 80 or 100 µg/ml in the absence or presence of Z-VAD-fmk (10 µM, InVivoGen, USA). Following NP exposure, cells were washed and stained with cell death dye as described above and analyzed for cell death.

### Macrophage polarization studies

RAW 264.7 macrophages were seeded at 1*10^5^ cells/flask in 25 cm^2^ cell culture flasks and allowed to settle overnight in a humidified atmosphere at 37 °C and 5% CO_2_. Media were then removed and fresh media (5 ml) was given with 33% Cu-doped TiO_2_ NPs at 40 µg/ml for 24 h. Media were removed, cells were washed with PBS, trypsinized and all supernatants, washing media and cells were added together and centrifuged at 2500 rpm for 6 min after which the cells were split into 2 fractions and one half was stained with anti-CD163 antibody (SB436-anti-CD163 clone TNKUPJ, Thermo Fisher Scientific) and anti-CD206 antibody (APC-anti-206 clone MR6F3 Thermo Fisher Scientific) and the other half was stained with anti-CD86 antibody (FITC-anti-CD86 clone GL1, Thermo Fisher Scientific) and anti-MHC II antibody (PE-Cy7-anti-MHC-II clone M5-114-15-2, Thermo Fisher Scientific) for 30 min at 4 °C. The cells were then centrifuged again, and resuspended in PBS after which they were run on the ImageStream X Mark II (Merck, Belgium) for analysis. Using iDEAS software, focused and single cells were selected and analysed for expression levels of the different markers.

### Gene expression analysis

For gene expression studies, the following RT-PCR gene pathway array was used: the murine stress and toxicity responses array (PAMM-003Z, Qiagen Benelux BV, Netherlands). These experiments were conducted as described previously. In short, cells were seeded at 1.5*10^5^ cells/mL and allowed to settle overnight. Then, cells were either left untreated (controls) or incubated for 24 h with 33% Cu-doped TiO_2_ NPs at 20 µg/ml. Next, cells were washed twice with PBS and harvested into centrifuge tubes. RNA was extracted using the Qiagen RNeasy Mini Kit (Qiagen, Benelux BV, Netherlands) according to the manufacturer's instructions. Each RNA sample underwent DNase digestion to eliminate genomic DNA contamination using the RNase-Free DNase Set (Qiagen, Benelux BV, Netherlands). RNA samples were converted into first strand cDNA using the RT2 First Strand Kit (Qiagen Benelux BV, Netherlands) where Genomic DNA Elimination Mixture was applied according to the manufacturer's instructions and samples were PCR amplified. First strand cDNA was then used in the RT-PCR after samples were added to the RT2qPCR Master Mix plus SybrGreen (Qiagen Benelux BV, Netherlands). RT-PCR was performed on the iCycler iQ5 Thermal Cycler (Bio-Rad Laboratories N.V., Belgium). PCR array data was analyzed using the ∆∆Ct method via the GeneGlobe web portal (https://geneglobe.qiagen.com/us/analyze).

### In vitro DC experiments

#### De-immortalization of DCs

The immortalized DCs were de-induced (in the absence of dexamethasone/doxycyclin) for 3 days as described in Richter et al.[43] prior to performing any experiments.

#### Evaluation of surface markers upon NP exposure

De-immortalized DCs were seeded at 1*10^5^ cells/flask in 25 cm^2^ cell culture flasks and allowed to settle overnight in a humidified atmosphere at 37 °C and 5% CO_2_. Media were then removed and fresh media (5 ml) was given with 33% Cu-doped TiO_2_ NPs at 40 µg/ml for 24 h. As controls, cells were exposed to the NPs in the media in the absence or presence of 1 µM MCC950 (InvivoGen). Media were removed, cells were washed with PBS, trypsinized and all supernatants, washing media and cells were added together and centrifuged at 2500 rpm for 6 min after which cells were stained with anti-CD86 antibody (FITC-anti-CD86 clone GL1, Thermo Fisher Scientific), anti-MHC II antibody (PE-Cy7-anti-MHC-II clone M5-114–15-2, Thermo Fisher Scientific) and anti-CD80 antibody (APC-anti-CD80 clone 16-10A1, Thermo Fisher Scientific) for 30 min at 4 °C. The cells were then centrifuged again, and resuspended in PBS after which they were run on the ImageStream X Mark II (Merck, Belgium) for analysis. Using iDEAS software, focused and single cells were selected and analysed for expression levels of the different markers.

#### Evaluation of NLRP3 expression, IL1β, IL12 or IL23 of DCs upon NP exposure

De-immortalized DCs were seeded at 1*10^5^ cells/flask in 25 cm^2^ cell culture flasks and allowed to settle overnight in a humidified atmosphere at 37 °C and 5% CO_2_. Media were then removed and fresh media (5 ml) was given with 33% Cu-doped TiO_2_ NPs at 40 µg/ml for 24 h. As controls, cells were exposed to the NPs in the media in the absence or presence of 1 µM MCC950 (InvivoGen). Cell media were then collected, concentrated using Pierce® protein concentrators followed by ELISA for murine IL1β (AMSBIO, code AMS.EM0109-CM, Abingdon, UK), IL12 (AMSBIO, code AMS.ELK9395, Abingdon, UK), IL23 (AMSBIO, code AMS.T3094, Abingdon, UK). DCs were then lysed in (5 ×) Laemmli SDS buffer supplemented with 1% β-mercaptoethanol, 0.01% bromophenol blue and protease inhibitor cocktail (Roche). Equal amounts of protein were analyzed by SDS-PAGE. Proteins were transferred to PVDF membranes and incubated with anti-NLRP3 antibody (1:1000, Cryo-2; Adipogen) and anti-GAPDH (1:5000; Cell Signaling). Secondary HRP-linked antibodies against rabbit and mouse IgG (1:2000) were from Cell Signaling. Immunoblots were imaged using the enhanced chemiluminescence reagent (Thermo Scientific) and visualized using a BIORAD camera (Universal Hood II). GAPDH was used as loading control.

#### Evaluation of DC viability upon NP exposure

De-immortalized DCs were seeded at 1*10^5^ cells/flask in 25 cm^2^ cell culture flasks and allowed to settle overnight in a humidified atmosphere at 37 °C and 5% CO_2_. Media were then removed and fresh media (5 ml) was given with 33% Cu-doped TiO_2_ NPs at 40 µg/ml for 24 h. Media and cells were collected by trypsinization and scraping, after which the samples were centrifuged at 2500 rpm for 6 min and cells were stained with propidium iodide (PI) for 10 min prior to being washed, centrifuged and resuspended in PBS after which they were run on the ImageStream X Mark II (Merck, Belgium) for analysis on dead cells.

#### Evaluation of DC activity surface markers upon tumor cell supernatants exposure

E0771 tumor cells, which are syngeneic to the conditionally immortalized DCs, were seeded at 1*10^5^ cells/flask in 25 cm^2^ cell culture flasks and allowed to settle overnight in a humidified atmosphere at 37 °C and 5% CO_2_. Media were then removed and fresh media (5 ml) was given with 33% Cu-doped TiO_2_ NPs at 40 µg/ml for 8 h. Media were then removed and cells were washed extensively to remove any free NPs, after which the cells were kept in culture for another 16 h. The supernatant of these cells or control tumor cells (not exposed to any NPs) was then collected and supplemented (1/1 ratio) with full DC media after which the DCs (seeded at 1*10^5^ cells/flask in 25 cm^2^ cell culture flasks) were allowed to grow for 24 h in full media supplemented with an equal amount of tumor cell supernatant. Media were removed, cells were washed with PBS, trypsinized and all supernatants, washing media and cells were added together and centrifuged at 2500 rpm for 6 min after which cells were stained with anti-CD86 antibody (FITC-anti-CD86 clone GL1, Thermo Fisher Scientific) and anti-CD80 antibody (APC-anti-CD80 clone 16-10A1, Thermo Fisher Scientific) for 30 min at 4 °C. The cells were then centrifuged again, and resuspended in PBS after which they were run on the ImageStream X Mark II (Merck, Belgium) for analysis. Using iDEAS software, focused and single cells were selected and analysed for expression levels of the different markers.

#### Evaluation of activated DCs in view of T cell polarization and activation

De-immortalized DCs were seeded at 1*10^5^ cells/flask in 25 cm^2^ cell culture flasks and allowed to settle overnight in a humidified atmosphere at 37 °C and 5% CO_2_. Media were then removed and fresh media (5 ml) was given with either no additions (controls), 33% Cu-doped TiO_2_ NPs at 40 µg/ml (NP condition) or addition of maturation factors IL1β (25 ng/ml) TNFα (50 ng/ml) and IFNγ (1000 units/ml) (all from PeproTech; this is the “classical” activation scheme) for 24 h. The DCs were pulsed with OVA protein and were subsequently incubated with freshly isolated syngeneic (C57Bl6 splenocyte-derived) CD3^+^ T cells for 8 h. Media were then removed, cells were centrifuged at 2500 rpm for 6 min after which cells were stained with anti-CD4 antibody (FITC-anti-CD4, Thermo Fisher Scientific) and anti-CD8 antibody (APC-anti-CD8, Thermo Fisher Scientific) and anti-CD3 antibody (PE-anti-CD3, Thermo Fisher Scientific) for 30 min at 4 °C. The cells were then centrifuged again, and resuspended in PBS after which they were run on the ImageStream X Mark II (Merck, Belgium) for analysis. Using iDEAS software, focused and single cells were selected and analysed for expression levels of the different markers. T cells were also exposed to SIINFEKL peptide (OVA-derived) and plated on ELISPOT plates for detection of granzyme B (EL1865, R&D Systems) while ELISA was performed for the detection of perforin (kit NBP3-00,452, NovusBio).

#### Evaluation of DC activation levels

De-immortalized DCs were seeded at 1*10^5^ cells/flask in 25 cm^2^ cell culture flasks and allowed to settle overnight in a humidified atmosphere at 37 °C and 5% CO_2_. Media were then removed and fresh media (5 ml) was given with either no additions (controls), 33% Cu-doped TiO_2_ NPs at 40 µg/ml (NP condition) or addition of maturation factors IL1β (25 ng/ml) TNFα (50 ng/ml) and IFNγ (1000 units/ml) (all from PeproTech; this is the “classical” activation scheme) for 24 h. Media were then removed and cells were incubated with soluble recombinant CD40L (BioTechne) at 16 µg/ml for 24 h after which the supernatants was collected and used to determine IL12p70 levels by ELISA (M1270, R&D Systems). Cells were centrifuged at 2500 rpm for 6 min after which cells were stained with anti-Ccr7 antibody (PE-anti-CD197 (Ccr7), clone 4B12, Thermo Fisher Scientific) for 30 min at 4 °C. The cells were then centrifuged again, and resuspended in PBS after which they were run on the ImageStream X Mark II (Merck, Belgium) for analysis.

#### Evaluation of DC migration levels

De-immortalized DCs were seeded at 1*10^5^ cells/flask in 25 cm^2^ cell culture flasks and allowed to settle overnight in a humidified atmosphere at 37 °C and 5% CO_2_. Media were then removed and fresh media (5 ml) was given with either no additions (controls), NP conditions or classical activation scheme for 24 h. For migration, DCs (25*10^3^ in 25 µL volume were seeded on the membrane surface of 5 µm pore ChemoTx 96-well plate (NeuroProbe, Gaithersburg, MD) and incubated for 90 min at at 37 °C, with 10 ng/ml 6C-kine present in the bottom chamber, after which the number of migrated DCs in the bottom chambers were counted. To determine the IL-12p70-producing ability of migrated DCs, recombinant soluble CD40L was added directly to the bottom chambers, containing the migrated DCs, for 24 h, after which the IL12p70 was determined from the supernatant by ELISA (M1270, R&D Systems).

### Animal studies

Female DBA/2 mice (Harlan Laboratories, Cambridgeshire, UK), 5–7 weeks old, were used in this study. The animal studies used a syngeneic tumor model in which DBA/2 animals received 500,000 KLN 205 cells in 200 µl saline as a subcutaneous injection on the lower part of the left side of the back. For DC-based studies, C57Bl6 mice bearing syngeneic E0771 breast adenocarcinoma following subcutaneous engraftment in the lower part of the back were used. All mouse surgical procedures and imaging were performed with the animals anesthetized by inhalation of 2% isoflurane. The condition of the animals was monitored every day and their weight was measured every other day. Tumors were measured with calipers every other day. When tumors reached the size of minimally 50 mm^3^ (approximately 10–14 days after tumor inoculation), the animals were divided into different groups of similar tumor size for further experiments. When tumors became larger than 1.5 cm or a deep ulcer was formed, euthanasia was performed. If animal weight dropped by 10%, the animals were sacrificed. For the tumor growth studies, all animals were sacrificed 5 weeks after NP administration. For euthanasia, animals were subjected to 5% isoflurane inhalation. To ensure death following isoflurane inhalation cervical dislocation was performed. Tumors were then removed from the animals and used for ex vivo imaging. All animal studies were approved by KU Leuven’s Institutional Animal Care and Use Committee (IACUC; approval number P149/2019) in accordance with the principals and procedures outlined in national and European regulations.

### Luminescence imaging of KLN205 tumors

DBA/2 mice with fLuc-expressing KLN 205 tumors of minimally 50 mm^3^ were divided into different groups containing 8 animals per group. Before each imaging session, the mice were injected intraperitoneally with 126 mg/kg D-luciferin (Promega, Madison, WI, USA) dissolved in PBS (15 mg/ml). Next, all 4 animals per group were positioned in the IVIS Spectrum and images were acquired after 10 min under 2% isoflurane inhalation. The animals received a peritumoral injection of either 100 µl of saline (control animals) or 100 µl saline containing Cu-doped TiO_2_ NPs at 100 µg/animal. Additional studies included the use of mice treated with 33% Cu-doped TiO_2_ NPs in the presence of anti-CD8 antibody, anti-CSFR1 antibody or anti-PDL1 antibody (Bio X Cell, all antibodies given intravenously at 200 µg/mouse in 3 injections. For anti-CD8 and anti-CSFR1, at 5, 3 and 1 day prior to NP administration. For anti-PDL1 this was given at 2 days before, together with and 2 days following NP administration). Images were acquired just before NP or saline injection and after 4, 8, 12, 16 and 20 days post NP injection (medium binning, f stop = 1, time = 25 s). Bioluminescence images were analyzed using the LivingImage (Perkin Elmer, Waltham, MA) processing software. Regions of interest (ROIs) were drawn around the bioluminescent signals in the tumor regions of the mice, and measurements were generated as the total flux (p/s/cm^2^/sr) from the selected ROIs.

### Systemic effect of NP treatment

To evaluate the efficacy of the 33% Cu-doped TiO_2_ NPs on tumor growth, DBA2 mice were subcutaneously grafted with non-luminescent KLN205 tumors on both the left and right flanks. When tumors reached 50 mm^3^, animals received anti-PDL1 antibody (200 µg in 100 µL PBS; 3 injections, one every other day) by intravenous administration. At day 3 (second bolus of anti-PDL1 antibody), animals also received a single peritumoral administration of 33% Cu-doped TiO_2_ NPs (100 µg in 100 µl saline) in the right tumor. Tumor growth was then monitored by caliper measurements every day.

### Imagestream-based analysis of tumor, spleen and draining lymph node immune composition

In DBA2-tumor bearing animals, treated with Cu-doped TiO_2_ NPs, tumors, spleen and tumor-draining lymph node were removed from the animals 14 days following NP administration. The different organs were dissociated into single cells using the GentleMACS tissue dissociator and associated kits (tumor dissociation kit and murine spleen dissociation kit, respectively; Miltenyi Biotec, Gladbach, Germany). The tumor samples were cut into small pieces, transferred in gentleMACS C-tubes containing RPMI/DMEM media and kit enzymes and were broken down using the gentleMACS. After the organs were processed, the gentleMACS C-tubes were centrifuged 30’ on 1,5 rpm, the content of the gentleMACS C-tube was passed first through 70 µm, followed by 40 µm strainer and was centrifuged for 7’ on 300 g. The pellet was lysed using RBC lysis buffer for exactly 2’, centrifuged for 5’ on 300 g and resuspended in 1 mL media, which was then added slowly on top of 1 mL FBS to form a layer of media on top of the FBS. After centrifugation for 5’ on 100 g, the single cells inside the media sank to the bottom of the FBS and the single cells were separated from the debris. The supernatants was removed, the cells were washed with 1XPBS, centrifuged for 5’ on 1.4 rpm and were incubated with Fc Blocker (1:100, in 1XPBS + 1%FBS, Thermo Fischer Scientific, Ghent, Belgium) for 30’ on ice. The cells were washed with 1XPBS + 1%FBS, centrifuged for 5’ on 1.4 rpm and incubated with various antibody cocktails, depending on the sample for 1 h on ice, protected from light, where all the antibodies were diluted in 1XPS + 1%FBS.

For the tumor sample, the following antibodies were used: rabbit anti-mouse F4/80 (AF488, Cell Signaling Technologies), rabbit anti-mouse CD3 (PE, Cell Signaling Technologies), rabbit anti-mouse CD8 (APC, Cell Signaling Technologies). For spleen, the following antibodies were used: eFluor450-CD3, PE-CD19, FITC-F4/80, PE-Texas Red-CD45 (cocktail A, all from Thermo Fisher Scientific) and APC-CD4, FITC-CD8, PE-CD69, eFluor450-CD45 (cocktail B, all from Thermo Fisher Scientific). For the tumor-draining lymph nodes, following antibodies were used: PE-Cy7-CD11c and APC-MHC-II (from Thermo Fisher Scientific) for DC gating strategies and combined with AF488-CD172α, PE-XCR-1 (from BioLegend, Belgium) for subclassification into cDC1 and cDC2 families. For evaluation of Th17 responses, CD3^+^ T cells were isolated by magnetic beads (Miltenyi), fixed in 2% PFA and permeabilized (0.5% saponin in PBS with 1% BSA) after which the cells were stained intracellularly for 30 min with FITC-IL17A (clone eBio17B7), eFluor660-IL17F (clone eBIO18F10), Per-CP-eFluor710-IL22 (clone 1H8PWSR) and PE-IL21 (clone FFA21) all from Thermo Fisher Scientific.

The single cells were washed with 1XPBS + 1%FBS, resuspended in 1XPBS and transported in eppendorfs to the image-based cytometer Imagestream-X Mark II Imaging flow cytometer (Merck, Overijse, Belgium). Measurements were done by acquiring 50,000 single cells per sample using a magnification of 40 × in a 12-channel system. Compensation matrices for the different antibody cocktails were generated and used in post-acquisition analysis. For analysis, iDEAS software (Amnis Corporation, USA) was used, followed by FCS Express 7.0 for visualization. First, viable, focused and single cells were selected and gated, after which cell selections were gated based on the different markers used.

### Immunohistochemical staining of F4/80 and CD8

In DBA2 mice bearing KLN205 tumors, animals were sacrificed and tumors were removed without any NP injection (day 0; control) or 6, 12 or 18 days following peritumoral administration of 33% Cu-doped TiO_2_ NPs. Tumor samples were dehydrated after being fixed for minimum 48 h in 4% PFA at 4 °C. The tissues were after subsequently embedded in OCT compound (Sakura-Finetek, CA, USA) and frozen at − 80 °C. Tissue slices of 10 um were cut using the Cryostar NX70 (Thermo Fisher Scientific, Ghent, Belgium) and placed on glass microscope slides (Leica Frost slides). The tumor samples were air-dried at RT for 30’ without dehydration and were washed in 1X PBS for 5’. The samples were fixed in 100% cold MeOH for 6’ at − 20 °C, washed for 5’ in 1XPBS, and incubated for 15’ with Proteinase K (1:500, in 1XPBS) at 37 °C. After washing the slides 5’ with 1XPBS, the slides were blocked for 1 h at RT with 1XPBS + 10% normal goat serum (NGS) and 1%FBS and washed again twice with 1XPBS for 5’ each. The samples were blocked with Avidin (0.001%) for 20’, followed by washing with 1XPBS two times for 2’ each and blocked again with Biotin (0.001%) for 20’ and washed twice for 2’ in 1XPBS each. The samples were incubated with rat anti-F4/80 antibody (1:25, in 1XPBS + 1%NGS, Abcam, Cambidge, UK) overnight at 4 °C.

After leaving the samples for 20’ at RT, the samples were washed twice in 1XPBS for 5’ each, blocked for 20’ with hydrogen peroxidase 3%, washed twice in 1XPBS for 5’ each and incubated with anti-Rat-Biotin (1:300, in 1XPBS + 1%NGS, Jackson ImmunoResearch Europe Ltd, Ely, UK) at RT for 1 h. The samples were washed twice in 1XPBS for 5’ each, incubated with streptavidin-HRP (1:150, in 1XPBS + 1%NGS, Invitrogen, Thermo Fisher Scientific, Ghent, Belgium) for 30’ at RT, washed again two times in 1XPBS 5’ each and incubated with Alexa Fluor Tyramide 594 (1:100, Thermo Fisher Scientific, Ghent, Belgium) + hydrogen peroxidase 3% (1:100) + Tris Buffer HCl pH 7.4 (1:1) for 10’ at RT. The reaction was stopped using Stop Reagent (1:11, in 1XPBS, Alexa Fluor 594 Tyramide SuperBoost Kit, Invitrogen, ThermoFischer Scientific, Ghent, Belgium) for 2’ at RT, washed three times in 1XPBS for 5’ and incubated with rabbit anti-CD8 (1:200, in 1XPBS + 1%NGS, Sigma-Aldrich, Bornem, Belgium) overnight at 4 °C. The samples were washed twice in 1XPBS for 5’ each, incubated with Goat Anti-Rabbit IgG secondary antibody poly HRP (1:1, Thermo Fischer Scientific, Ghent, Belgium) 1 h at RT, washed twice in 1XPBS for 5’ each, incubated with Alexa Fluor Tyramide 488 (1:100, Thermo Fisher Scientific, Ghent, Belgium) + hydrogen peroxidase 3% (1:100) + Tris Buffer HCl pH 7.4 (1:1) for 10’ at RT, incubated after for 2’ at RT with Stop Reagent (1:11, in 1XPBS), washed three times in 1XPBS for 5’ each and incubated for 10’ at RT with DAPI (1:1000, in 1XPBS, Thermo Fisher Scientific, Ghent, Belgium). Finally, the samples were washed twice in 1XPBS for 5’ each, mounted with Fluoromont (Sigma-Aldrich, Merck Chemicals, Overijse, Belgium), air-dried for 30’ with dehydration and the cover slides were sealed with transparent nail polish. The slides were then imaged (40 × magnification) by a Vectra Polaris automated multispectral slide scanner system (Perkin Elmer, Life Sciences, Zaventem, Belgium) and analysed using QuPath. The images were automatically stitched together to cover the entire slide and were saved as.qptiff file format for further analysis.

### Blood biochemistry

DBA/2 mice with fLuc-expressing KLN 205 tumors of minimally 50 mm^3^ were divided into different groups containing 6 animals per group. Animals were treated with either the different Cu-doped TiO_2_ NPs at 100 µg/mouse. Blood samples were collected retroorbitally following animal sacrifice (200 µl/animal), and samples were collected and centrifuged in heparin-containing tubes to separate plasma from serum (15 min at 3500 rpm). Next, 75 µl serum was added on analysis discs (Samsung Comprehensive test 16 V) enabling analysis of 16 different markers using the Samsung PT10V chemistry analyzer (SCIL Animal care company GmbH, Viernheim, Germany). The following markers were analyzed: glucose, urea, creatinine, urea/creatinine ratio, phosphates, calcium, total protein, albumin, globulin, albumin-globulin ratio, alanine aminotransferase, alkaline phosphatases, bilirubin, cholesterol, triglycerides and amylase.

### Macroscopic analysis (H&E)

For all control and treated animals, after 10 days following vehicle or NP administration, tumors and major organs (lung, liver, heart, kidney) were isolated and dehydrated after being fixed for minimum 48 h in 4% PFA at 4 °C. The tissues were after subsequently embedded in OCT compound (Sakura-Finetek, CA, USA) and frozen at -80 °C. Tissue slices of 10 um were cut using the Cryostar NX70 (Thermo Fisher Scientific, Ghent, Belgium) and placed on glass microscope slides (Leica Frost slides). The tumor samples were air-dried at RT for 30’ without dehydration and were washed in 1X PBS for 5’, stained with hematoxylin for 3 min and protected from light. Then, they were washed with Milli-Q water (5 min), 80% ethanol-0.15% HCl (1 min) and Milli-Q water (5 min), respectively. Next, sections were washed for 30 s with ammonia water 0.3% v/v. Next, the samples were washed again with Milli-Q water for 5 min and washed once with ethanol 95% for 1 min. After washing with ethanol, sections were stained for 1 min with eosin and protected from light. In the following, sections were dehydrated once with ethanol 95% and two times with ethanol 100% (5 min) and cleared two times with pure xylene (5 min). Finally, sections were mounted with DPX and analyzed under light microscope to assess the systemic toxicity of each treatment group compared to the control. Further, the images were analyzed using QuPath software.

### Evaluation of antibody responses in vivo on DC vaccination

De-immortalized DCs were seeded at 1*10^5^ cells/flask in 25 cm^2^ cell culture flasks and allowed to settle overnight in a humidified atmosphere at 37 °C and 5% CO_2_. Media were then removed and fresh media (5 ml) was given with either no additions (controls), NP conditions or classical activation scheme for 24 h. Cells were then pulsed with OVA protein and pooled together per conditions. Next, cells were administered intravenously in C57Bl6 mice twice with 2*10^6^ cells per mouse in 100 µl PBS with a 10-day interval between injections. Another 10 days later, animals received a subcutaneous administration of 3 µg OVA protein in Freund’s complete adjuvant. Serum was then taken 7 days following administration of the OVA booster and stored at -20 °C. High-binding microtiter plates (Greiner) were coated with OVA (20 µg/ml in PBS) overnight. Threefold serial dilutions of sera were incubated for 90 min in PBS containing 0.1% bovine serum albumin. After this, peroxidase-conjugated anti-mouse IgG (1/3,000), IgG1 (1/1,000), or IgG2a (1/1,000) (all obtained from Southern Biotechnology) was added for 90 min at room temperature. The reaction was developed with 100 µl of a 1-mg/ml solution of *o*-phenylenediamine dihydrochloride (Sigma) in 0.1 M citrate buffer (pH 4.5) containing 0.04% H_2_O_2_, and the results were read at 450 nm.

Splenocytes were isolated and single cell suspensions were analyzed for OVA-specific IFNy production. In brief, isolated splenocytes were seeded at different cell densities into Iscoves medium containing 5% horse serum in the presence or absence of OVA (500 µg/ml) and incubated at 37 °C for 24 h in anti-IFN-γ-coated flat-bottom 96-well plates. The cells were removed by extensive washing, and biotinylated anti-mouse IFN-γ was added overnight at 4 °C; this was followed by 45 min of incubation with 2 µg of peroxidase-labeled avidin (Sigma) per ml at room temperature. Color was developed with 100 µl of peroxidase substrate containing 3,3′, 5,5′-tetramethylbenzidine (0.1 mg/ml; Sigma) and 0.06% H_2_O_2_ in 0.05 M phosphate-citrate buffer at pH 5.0. The reaction was stopped by adding 25 µl of 1 M H_2_SO_4_, and the absorbance at 450 nm was determined.

### Evaluation of DC-mediated antitumor efficacy

C57Bl6 mice received a subcutaneous graft of 1*10^6^ OVA-expressing E0771 cells and tumors were allowed to develop. De-immortalized DCs were seeded at 1*10^5^ cells/flask in 25 cm^2^ cell culture flasks and allowed to settle overnight in a humidified atmosphere at 37 °C and 5% CO_2_. Media were then removed and fresh media (5 ml) was given with either no additions (controls), NP conditions or classical activation scheme for 24 h. Cells were then pulsed with OVA protein and pooled together per conditions. Next, DCs cells were administered intravenously (2*10^6^ cells per mouse in 100 µl PBS) in the OVA-E0771 tumor-bearing mice at 3 days post tumor grafting and again one week later (10 days following original tumor grafting). Tumor growth was then evaluated using caliper measurements for a period of up to 23 days. After that time, tumors and spleens were isolated and single cell suspensions were made. For the tumor samples, CD8^+^ T cells were isolated using FACS sorting and the level of granzyme B was determined by ELISPOT, while the level of perforin was determined by ELISA. From the spleen, CD8^+^ T cells were isolated by FACS and stained with OVA-specific MHCI dextramer-APC (Immudex) diluted at 1:2 and analysed using ImageStreamX Mark II analysis.

### Statistical analysis

All statistical analysis were performed using GraphPad 9 analytical software using one-way ANOVA to compare the difference from any treatment group to the control, unless otherwise indicated in the respective figure legend.

## Supplementary Information

**Additional file 1: Figure S1.** Histograms displaying the relative effect of the different nanoformulations on cell viability (left column), cell area (2nd column), cell aspect ratio (3rd column) and mitochondrial health (right column) for A549 cells (top row), KLN-205 cells (middle row) and Beas-2B cells (bottom row). All data are presented as mean + SEM and are expressed relative to untreated control cells (= 100%). The degree of significance is indicated where appropriate. In case only one condition is indicated, then the level of significance is maintained for any subsequent time point unless otherwise indicated. (NS: not significant; p < 0.05: *, p < 0.01: **; p < 0.001: ***) based on ANOVA testing using GraphPad Prism 9 (*n* = 4). **Figure S2.** Histograms displaying the relative ROS (top row) and relative viability (bottom row) of KLN-205 (left column) and A549 (right column) exposed to pure CuO or 33% Cu-doped TiO_2_ in the presence or absence of the ROS-scavenger NAC. All data are presented as mean + SEM and are expressed relative to untreated control cells (= 100%). The degree of significance is indicated where appropriate; (p < 0.05: *, p < 0.01: **; p < 0.001: ***; p < 0.0001) based on ANOVA testing using GraphPad Prism 9 (*n* = 4).

## Data Availability

All data needed to evaluate the conclusions in the paper are present in the paper and/or the Supplementary Materials. The raw data on tumor characterization and NM delivery efficacy can be provided by the corresponding author pending scientific review and a completed material transfer agreement. Requests for the raw numerical data should be submitted to: s.soenen@kuleuven.be.
