# Supplementary material for: Cu-doped TiO2 nanoparticles improve local antitumor immune activation and optimize dendritic cell vaccine strategies
Source: J Nanobiotechnology. 2023 Mar 13;21:87. doi: 10.1186/s12951-023-01844-z (PMC10009859; doi:10.1186/s12951-023-01844-z)
Supplement: Supplementary file 1 — Additional file 1: Figure S1. Histograms displaying the relative effect of the different nanoformulations on cell viability (left column), cell area (2nd column), cell aspect ratio (3rd column) and mitochondrial health (right column) for A549 cells (top row), KLN-205 cells (middle row) and Beas-2B cells (bottom row). All data are presented as mean + SEM and are expressed relative to untreated control cells (= 100%). The degree of significance is indicated where appropriate. In case only one condition is indicated, then the level of significance is maintained for any subsequent time point unless otherwise indicated. (NS: not significant; p < 0.05: *, p < 0.01: **; p < 0.001: ***) based on ANOVA testing using GraphPad Prism 9 (n = 4). Figure S2. Histograms displaying the relative ROS (top row) and relative viability (bottom row) of KLN-205 (left column) and A549 (right column) exposed to pure CuO or 33% Cu-doped TiO2 in the presence or absence of the ROS-scavenger NAC. All data are presented as mean + SEM and are expressed relative to untreated control cells (= 100%). The degree of significance is indicated where appropriate; (p < 0.05: *, p < 0.01: **; p < 0.001: ***; p < 0.0001) based on ANOVA testing using GraphPad Prism 9 (n = 4). [file 12951_2023_1844_MOESM1_ESM.docx]

Cu-doped TiO_2_ nanoparticles improve local antitumor immune activation and optimize dendritic cell vaccine strategies.

Evelien Hesemans,^1†^ Neshat Saffarzadeh,^1†^ Christy Maksoudian,^1^ Mukaddes Izci,^1^ Tianjiao Chu,^1^ Carla Rios Luci,^1^ Yuqing Wang,^2,3^ Hendrik Naatz,^2,3^ Sebastian Thieme,^4^ Cornelia Richter,^4^ Bella B. Manshian,^5,6^ Suman Pokhrel,^2,3^ Lutz Mädler,^2,3^ Stefaan J. Soenen^1,6,7*^.

**Affiliations**

^1^NanoHealth and Optical Imaging Group, Department of Imaging and Pathology, KULeuven, Belgium.

^2^Leibniz Institute for Materials Engineering IWT, Badgasteiner Straße 3, D-28359 Bremen, Germany

^3^Faculty of Production Engineering, University of Bremen, Badgasteiner Straße 1, D-28359 Bremen, Germany

^4^UniversitatsKlinikum Dresden, Germany.

^5^Translational Cell and Tissue Research Unit, Department of Imaging and Pathology, KULeuven, Belgium

^6^Leuven Cancer Institute, KULeuven, Belgium

^7^KU Leuven Institute of Physics-based Modeling for In Silico Health, KULeuven, Belgium

^†^These authors contributed equally to this work

^*^Corresponding author email: s.soenen@kuleuven.be

**Supporting information**


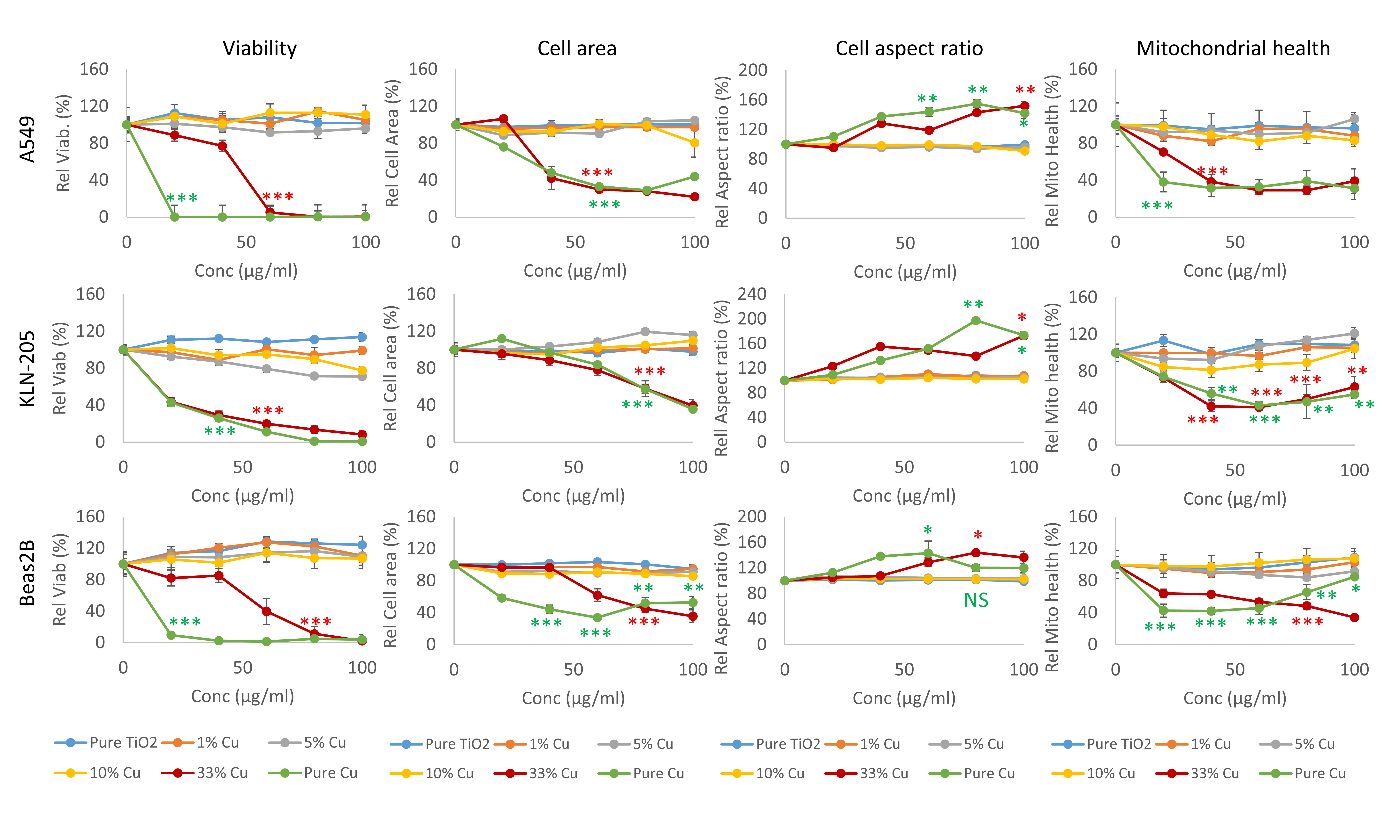


**Figure S1.** Histograms displaying the relative effect of the different nanoformulations on cell viability (left column), cell area (2^nd^ column), cell aspect ratio (3^rd^ column) and mitochondrial health (right column) for A549 cells (top row), KLN-205 cells (middle row) and Beas-2B cells (bottom row). All data are presented as mean + SEM and are expressed relative to untreated control cells (= 100%). The degree of significance is indicated where appropriate. In case only one condition is indicated, then the level of significance is maintained for any subsequent time point unless otherwise indicated. (NS: not significant; p < 0.05: *, p < 0.01: **; p < 0.001: ***) based on ANOVA testing using GraphPad Prism 9 (*n* = 4).


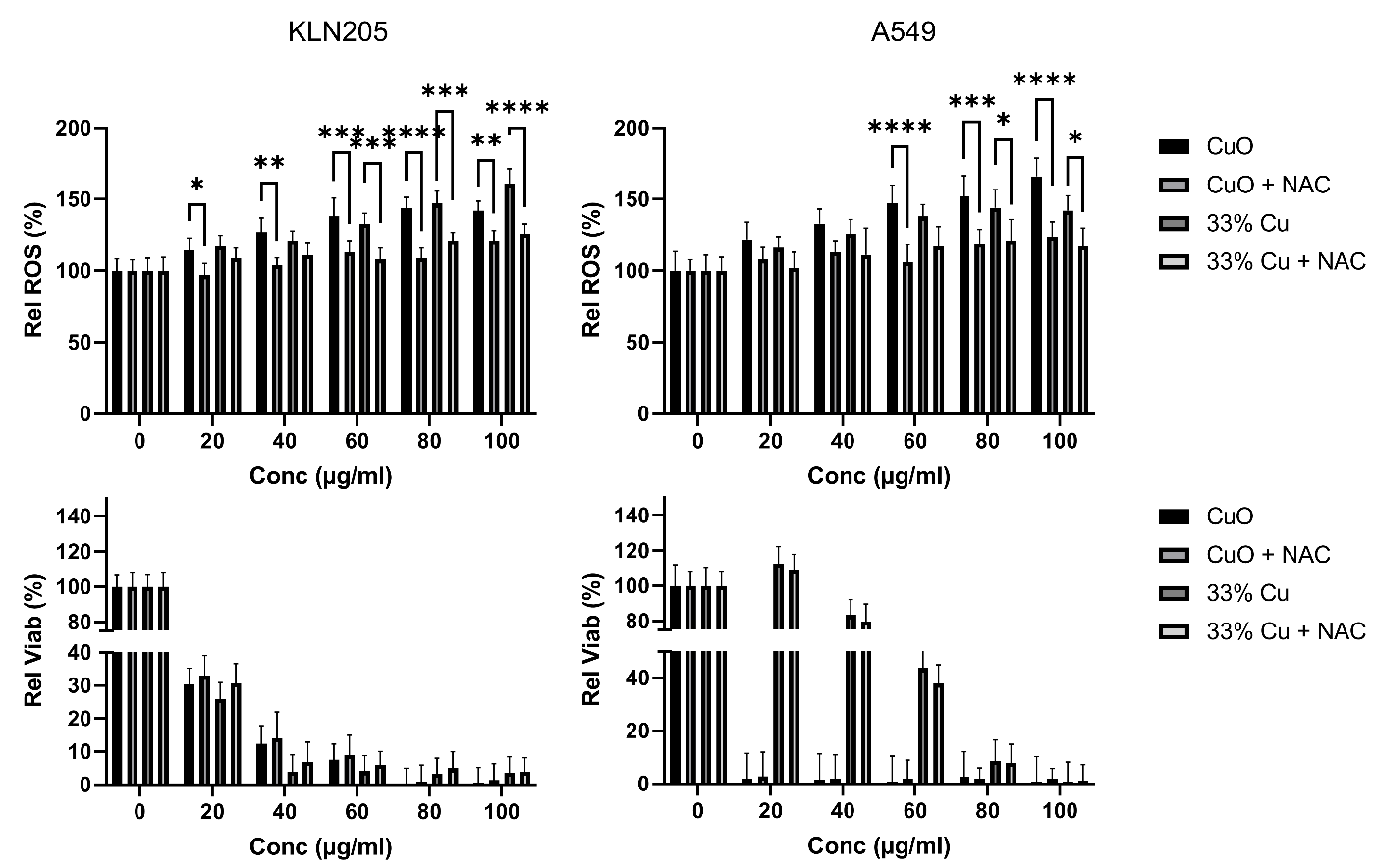
**Figure S2.** Histograms displaying the relative ROS (top row) and relative viability (bottom row) of KLN-205 (left column) and A549 (right column) exposed to pure CuO or 33% Cu-doped TiO_2_ in the presence or absence of the ROS-scavenger NAC. All data are presented as mean + SEM and are expressed relative to untreated control cells (= 100%). The degree of significance is indicated where appropriate; (p < 0.05: *, p < 0.01: **; p < 0.001: ***; p < 0.0001) based on ANOVA testing using GraphPad Prism 9 (*n* = 4).
